# Harnessing large vision and language models in agriculture: a review

**DOI:** 10.3389/fpls.2025.1579355

**Published:** 2025-09-02

**Authors:** Hongyan Zhu, Shuai Qin, Min Su, Chengzhi Lin, Anjie Li, Junfeng Gao

**Affiliations:** ^1^ Guangxi Key Laboratory of Brain-inspired Computing and Intelligent Chips, School of Electronic and Information Engineering, Guangxi Normal University, Guilin, China; ^2^ Key Laboratory of Integrated Circuits and Microsystems (Guangxi Normal University), Education Department of Guangxi Zhuang Autonomous Region, Guilin, China; ^3^ Department of Computer Science, University of Aberdeen, Aberdeen, United Kingdom

**Keywords:** large model, agriculture, natural language processing, computer vision, multimodal model

## Abstract

**Introduction:**

Agriculture is a cornerstone of human society but faces significant challenges, including pests, diseases, and the need for increased production efficiency. Large models, encompassing large language models, large vision models, and multimodal large language models, have shown transformative potential in various domains. This review aims to explore the potential applications of these models in agriculture to address existing problems and improve production.

**Methods:**

We conduct a systematic review of the development trajectories and key capabilities of large models. A bibliometric analysis of literature from Web of Science and arXiv is performed to quantify the current research focus and identify the gap between the potential and the application of large models in the agricultural sector.

**Results:**

Our analysis confirms that agriculture is an emerging but currently underrepresented field for large model research. Nevertheless, we identify and categorize promising applications, including tailored models for agricultural question-answering, robotic automation, and advanced image analysis from remote sensing and spectral data. These applications demonstrate significant potential to solve complex, nuanced agricultural tasks.

**Discussion:**

This review culminates in a pragmatic framework to guide the choice between large and traditional models, balancing data availability against deployment constraints. We also highlight critical challenges, including data acquisition, infrastructure barriers, and the significant ethical considerations for responsible deployment. We conclude that while tailored large models are poised to greatly enhance agricultural efficiency and yield, realizing this future requires a concerted effort to overcome the existing technical, infrastructural, and ethical hurdles.

## 1 Introduction

The significance of agriculture in the global economy is increasing steadily, and there is growing awareness regarding its sustainability. Ahirwar et al. (2019) believe that it is necessary to increase global agricultural food production by a minimum of 70% to meet the needs of the increasing world population. Unfortunately, there are many factors in agriculture that make it difficult to steadily increase grain production, including 1): crop diseases caused by pathogens such as bacteria, fungi, and viruses. These diseases can spread rapidly, often leading to devastating effects on entire crops. For instance, bacterial blight in rice and late blight in potatoes can wipe out significant portions of harvests. The economic impact is staggering, as farmers face not only reduced yields but also increased costs associated with disease management; 2): poor seed quality can lead to weak plant growth, reduced yields, and greater susceptibility to both diseases and pests. Farmers who use low-quality seeds often experience crop failures, which not only jeopardizes their income but also contributes to broader food insecurity within communities. Transitioning to certified, high-quality seeds is essential for improving crop resilience and productivity; 3): many agricultural tasks remain inefficient and labor-intensive, hindering productivity. Traditional methods of weeding, planting, watering, and harvesting are often time-consuming and can lead to resource wastage. For example, manual weeding not only consumes labor but may also fail to effectively control weed populations, resulting in reduced crop yields. The adoption of mechanization and modern farming techniques, such as precision agriculture (PA), can significantly improve efficiency.

PA is an agricultural management approach that utilizes modern technology to enhance production efficiency and sustainability. It encompasses sensor technology, unmanned aerial/ground vehicles (UAVs/UGVs), remote sensing technology, automation equipment, big data, machine learning (ML), and deep learning (DL) (Tokekar et al., 2016; Khanal et al., 2020; Saleem et al., 2021). This enables farmers to reduce production costs and improve decision-making capabilities, providing significant economic and social benefits. For crop diseases, traditional detection methods like polymerase chain reactions based on unique deoxyribonucleic acid sequences of pathogens, enzyme-linked immunosorbent assays on the basis of pathogens proteins and hyperspectral imaging, are constrained by their operational complexity and the requirement for bulky instruments (Yao et al., 2024). For selecting high-quality seeds, quality assurance programs employ various ways to attest seed quality attributes, including germination and vigor tests (ElMasry et al., 2019). But these methods have limitations in terms of time overhead, subjectivity, and the destructive nature of assessing seed quality (Medeiros et al., 2020). For a general tasks in agriculture, the use of pesticides for weed control may have negative impacts on the environment, and Phytotoxicity reactions can lead to diminished crop quality and reduced yields (Visentin et al., 2023). And the traditional solutions to these tasks are also inefficient due to these manned implements are dreadfully slow. On the other hand, driven by growing health consciousness, the public has long been worried about the safety and quality of food, which is linked to agricultural products. Reducing food losses and improving food safety rely significantly on the continuous monitoring of crop quality, especially the inspection of diseases during crop growth stage (Karthikeyan et al., 2020).

DL technologies in PA can effectively address the limitations of traditional methods by leveraging their powerful data processing and pattern recognition capabilities. For instance, DL can analyze vast amounts of data from sensors, drones, and satellite imagery to accurately identify crop health, soil characteristics, and potential diseases and pests (Nasir et al., 2021; Bouguettaya et al., 2022). This application enables farmers to obtain real-time insights, allowing for more scientifically informed management decisions, optimized resource use, and increased crop yields. However, DL technologies also have their limitations, primarily due to the high demand for model training (Sun et al., 2017). DL models typically require large amounts of labelled data to train and often need to be retrained when faced with new agricultural environments or crop varieties (Thenmozhi and Reddy, 2019). This repetitive training process is not only time-consuming but also requires significant computational resources and expertise. The effectiveness of transfer learning lies in its ability to apply models trained in one domain to a related domain, thus reducing the need for new datasets (Bosilj et al., 2020; Paymode and Malode, 2022). However, the diversity and complexity of agricultural environments can limit the effectiveness of transfer learning (Raffel et al., 2020). For example, differences in soil conditions, climate variations, and crop growth characteristics across regions can result in models trained in one area performing poorly in another. Therefore, although DL holds tremendous potential in PA, its adaptability and generalizability must be carefully considered to ensure that models remain effective in the ever-evolving agricultural field.

Large models are fundamentally distinguished from conventional DL models by their vast parameter counts (often billions) and extensive pre-training on massive, diverse datasets. By being exposed to a rich array of information, these models can better understand and adapt to various contexts, making them highly versatile tools in fields such as natural language processing (NLP), computer vision (CV), and decision-making (Kung et al., 2023). Crucially, unlike traditional DL models, large models develop “emergent abilities”—such as few-shot/zero-shot learning, complex reasoning, and strong generative abilities—that are not simply scaled-up versions of prior performance (Bommasani et al., 2021; Zhao et al., 2023c). As an efficient analytical means, large model, has found extensive application in the agricultural sector (Stella et al., 2023; Yang et al., 2023c). They have demonstrated excellent performance in analyzing agricultural data, pest and disease management, PA, and more. However, they still face many problems such as difficulty in obtaining agricultural data (Lu and Young, 2020), low model training efficiency, distribution shift (Chawla et al., 2021), and plant blindness (Geitmann and Bidhendi, 2023). In response to the challenges faced by traditional agriculture, we committed to conducting a comprehensive analysis of large models. First, we systematically summarized the history of large models, their applications in other fields, and their significance for agriculture. Subsequently, we introduced many applications of large models in agriculture. Furthermore, recognizing that large models were a relatively new technological approach, we outlined some solutions from ethical and responsibility perspectives. Finally, we summarized the current challenges and future directions of large models and drew conclusions on their effectiveness in the agricultural domain.

## 2 Feasibility analysis of large models in agriculture

Artificial intelligence (AI), whose main purpose is to establish systems that learn and think like human (Holzinger et al., 2019), just like human language and visual abilities. At present, research on large models is also focused on NLP and CV. Next, large language model (LLM), large vision model (LVM) and multimodal large language model (MLLM) will be introduced in detail.

### 2.1 Evolution and key milestones of large models

#### 2.1.1 Development trajectories of large language models

**Figure 1:**
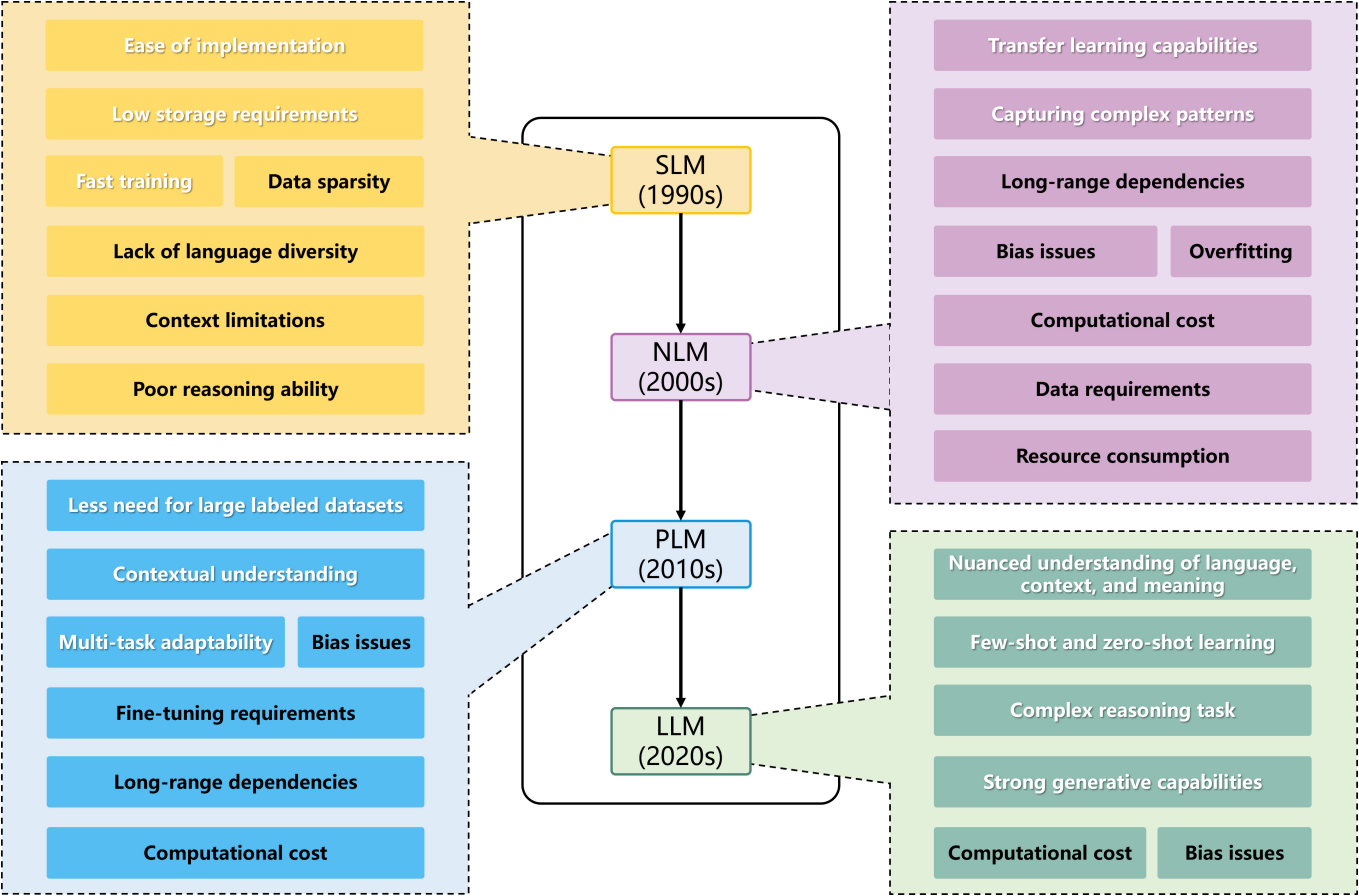
Development timeline of NLP models and their pros and cons. White characters represent advantages; black characters represent disadvantages.

LLM is a model based on NLP with a vast number of parameters (typically billions) trained on massive datasets of text and code, and we can divide the development of it into four stages (**Figure 1**):

1. Statistical Language Models (SLM): SLMs use traditional statistical methods (like n-grams) to learn word probabilities. Their effectiveness relies on the amount of data and estimation algorithms (Chelba et al., 2013). While SLMs are widely used in NLP, they have three main drawbacks: Scalability: Larger n requires more memory and parameters (n represents how many preceding words the model considers when predicting the next word); Information sharing: N-grams can’t share semantic information across similar words; Data sparsity: Techniques like data smoothing can help, but neural networks handle this better.
2. Neural Language Models (NLM): NLMs utilize various neural networks and are more effective than SLMs (Bengio et al., 2000; Mikolov et al., 2010; Sundermeyer et al., 2012). They address data sparsity using feedforward and recurrent neural networks (RNNs), which learn features automatically. Key developments include: Feedforward neural networks (FFNNLM): Proposed by Bengio et al. in 2003, they learn distributed word representations (Bengio et al., 2000); RNN Language Model (RNNLM): Introduced by Mikolov et al., but struggles with long-term dependencies (Mikolov et al., 2010). Long short-term memory (LSTM) networks were later added to overcome this (Sundermeyer et al., 2012).
3. Pre-trained Language Models (PLM): PLMs are categorized into feature-based and fine-tuning methods: Feature-based: Extracts features from large datasets (e.g., ElMo); Fine-tuning: Transfers entire model parameters to specific tasks, exemplified by BERT and GPT. Transformers, introduced by Google, employ a self-attention mechanism, facilitating better training and performance (Vaswani et al., 2017), GPT is fine-tuned from the Transformer. Due to the significant acceleration of model training by Transformer, it has gradually become the fundamental architecture for LLMs.
4. Large Language Models (LLM): LLMs have billions of parameters and exhibit unique capabilities, known as “emergent abilities”. Research shows that larger models perform better and are more sample-efficient. For instance, GPT-3 can generate expected outputs from input sequences without additional training, a feat beyond smaller models like GPT-2.

The transition from SLM to LLM signifies a progressive increase in model complexity, data handling abilities, and adaptability to tasks. Each new generation improves upon its predecessor to overcome limitations, fostering advancement in natural language processing. As shown in **Figure 1**, compared to other models, LLMs have a comprehensive understanding of language and excel at complex reasoning. Their strong few-shot, zero-shot, and generative capabilities allow them to adapt to new tasks with minimal examples. However, high computational costs and bias issues prevent them from being perfect. The high computational cost remains an unresolved challenge in today’s era of large data training. Bias issues can be mitigated through a series of review and regulatory measures, which will be detailed in section 4.

#### 2.1.2 Key advancements and capabilities of large vision models

**Figure 2:**
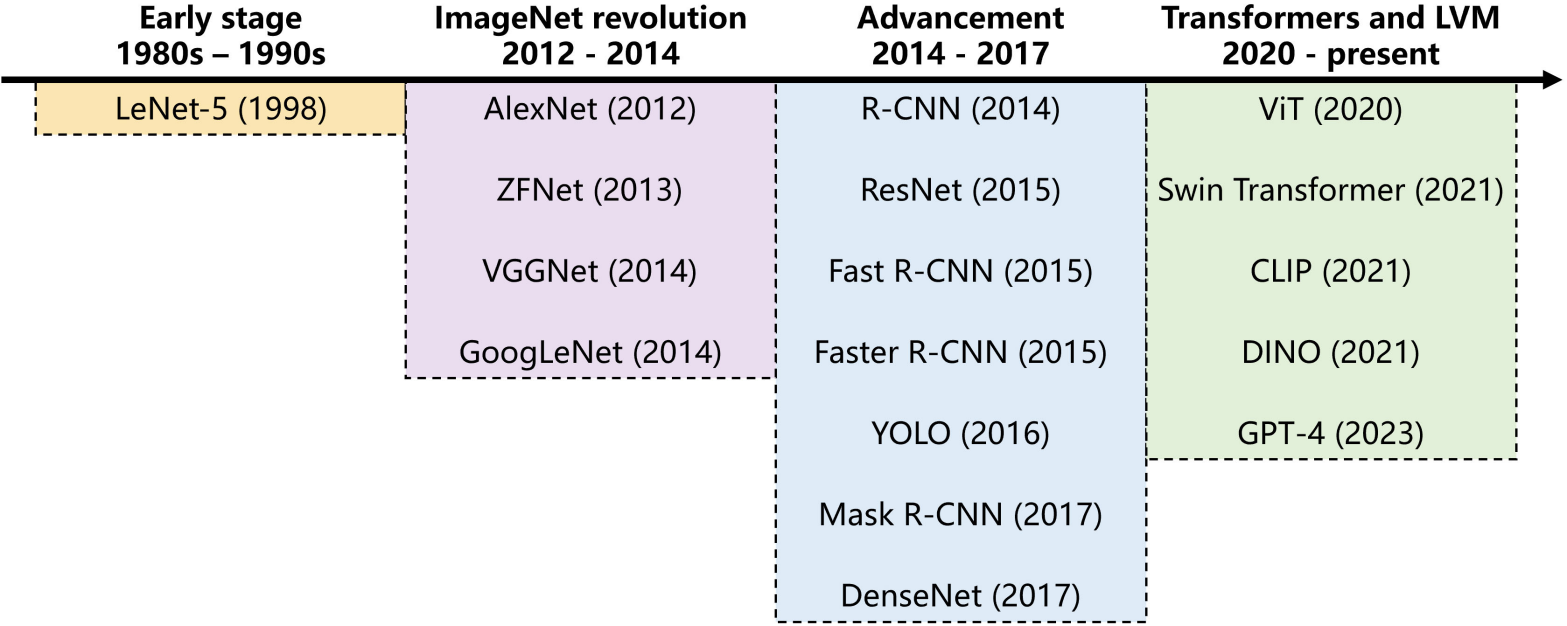
Development timeline of computer vision models.

LVMs are a new generation of models associated with CV, characterized by their immense scale and broad pre-training. Initially, LVM might have denoted purely vision-based models trained solely on image data. However, inspired by multimodal learning in LLM, the concept has evolved to include large models trained on both images and text, enabling rich cross-modal associations. CV models began their development in the 20th century and have continued to evolve significantly to the present day (**Figure 2**). Fueled by the availability of massive image datasets, the development of powerful DL architectures, and significant progress in large-scale pre-training techniques, LVMs have become one of the major development trends in CV models in recent years.

The research on CV models initially focused on shallow image feature extraction algorithms, including scale-invariant feature transform, histogram of oriented gradient, and other methods, but had significant limitations. In 2012, AlexNet (Krizhevsky et al., 2017) achieved a breakthrough success in ImageNet large scale visual recognition challenge, sparking a wave of convolutional neural networks (CNN) for vision models. With the development of DL, deep residual networks including VGGNet (Simonyan and Zisserman, 2014), GoogLeNet (Szegedy et al., 2015), and ResNet (He et al., 2016) were successively proposed, which improved the performance of image classification, object detection, semantic segmentation, etc. The boom of the Internet also enabled large-scale image datasets to be used for training vision models. Faster R-CNN (Ren et al., 2016), YOLO (Redmon et al., 2016), Mask R-CNN (He et al., 2017) emerged one after another.

In recent years, Transformer has been successfully applied in the domain of LVM, leading to the emergence of models like Vision Transformer (ViT) (Dosovitskiy et al., 2020) and DALL-E (Ramesh et al., 2021) which have garnered significant public attention. Unlike the traditional DL models mentioned above, LVMs such as ViT leverage transformer architectures and are typically pre-trained on significantly larger and more diverse datasets (e.g., billions of images). This foundational pre-training enables them to develop a more generalized understanding of visual concepts and emergent capabilities, allowing for superior performance on a wide range of downstream tasks, often with only limited domain-specific data. Their ability to grasp complex visual patterns and adapt to new conditions makes them highly versatile.

As detailed in **Table 1**, large vision models (LVMs) and traditional vision models differ significantly in their core architecture, data requirements, and capabilities. The fundamental distinction lies in their approach to context: Transformer-based LVMs leverage global self-attention to capture broad visual context and long-range dependencies, a significant leap from the local receptive fields of traditional convolutional models (Aymen et al., 2024; Malla et al., 2024). While this architectural shift grants LVMs superior generalization, it also introduces challenges like high computational demands and data hunger. Notably, research is actively addressing these limitations. For instance, Shi et al. (2024) proposed “Scaling on Scales (S²),” which enhances performance by increasing image scales rather than model size, providing new insights for the future development of vision models.

**Table 1:** Comparison of large vision models and traditional vision models.

Feature/Aspect	Large vision models	Traditional vision models
Core architecture	Transformer-based, global self-attention	Convolution-based, local receptive fields
Context & dependencies	Global context, excels at long-range dependencies	Local focus, struggles with long-range dependencies
Image handling	Processes image patches; more robust to variations	Uses sliding filters; sensitive to some variations
Data needs	Best with large-scale pre-training	Can work with smaller datasets, benefits from pre-training
Multimodal ability	Stronger, more inherent multimodal integration	Requires more specialized designs for multimodal
Parallelism	High (sequence processing)	Good for convolutions, poor for sequential tasks
Key advantages	Global understanding, long dependencies, scalability	Efficient local feature extraction
Key limitations	Can be data-hungry for pre-training	Limited global view, adversarial vulnerability

#### 2.1.3 The emergence of multimodal large language models

In addition to the LLMs and LVMs introduced above, MLLMs are also a research focus in the domain of AI. While LLMs perform well in text-based tasks, their capabilities alone cannot effectively reason about information presented in non-textual formats. Although LVMs perform well in the field of CV and possess some NLP abilities, researchers are not content with large models solely trained on text and images. MLLMs (Wu et al., 2023a) integrate multiple data types, such as images, text, language, audio, and more. It not only possesses the advantages of LLMs and LVMs, but also address the limitations of LLMs and LVMs by integrating multiple modalities, enabling a more comprehensive understanding of various data. It can be said that the developments in MLLMs have set up new avenues for AI, which make binary machines to understand and then process various data types (Wu et al., 2023a). For agriculture, MLLM allows tasks to no longer be confined to just images or text; instead, it can leverage both, and even utilize multimodal inputs like audio and video, breaking the limitations of images and text.

### 2.2 Current applications of large models in other domains

As shown in **Table 2**, many LLMs are designed to develop chatbots (BLOOM (Le Scao et al., 2023), PaLM2 (Anil et al., 2023), ERNIE 4.0) or complete NLP tasks, including text classification, machine translation, and sentiment analysis [OPT (Zhang et al., 2022b)]. Similarly, LVMs are primarily engineered to interpret and process visual information. They excel at core CV tasks such as image classification, object detection, segmentation, and image generation, often forming the foundation for systems needing to understand or interact with the visual world. Models like InternImage (Wang et al., 2023) and LLaVA (Liu et al., 2023b) represent efforts to enhance performance on complex visual analysis tasks, aiming to simulate and automate human visual processes.

**Table 2:** The currently popular and representative large models.

Original version	Latest version	Release date (original)	Release date (latest)	Types (original → latest)	Information	References
OPT	/	May 2^rd^, 2022	/	LLM	OPT promotes transparency, reproducibility, and broader community engagement and innovation in NLP research. (open source)	(Zhang et al., 2022b)
BLOOM	BLOOMZ	July 12^th^, 2022	December 15^th^, 2022	LLM	A decoder only model based on Transformer architecture. (open source)	(Le Scao et al., 2023)
PMC-LLaMA	/	Aprill 27^th^, 2023	/	LLM	Inject medical knowledge into existing LLM using 4.8 million biomedical academic papers. (open source)	(Wu et al., 2024)
PaLM2	/	May 11^st^, 2023	/	LLM	PaLM2 was a neural network-based language model that was considered one of the most advanced language models available at the time of its release in May 2023.	(Anil et al., 2023)
BloombergGPT	/	March 30^th^, 2023	/	LLM	A LLM for the financial field.	(Wu et al., 2023c)
OceanGPT-Basic-7B	OceanGPT-Basic-14B/7B/2B	October 3^rd^, 2023	July 4^th^, 2024	LLM	OceanGPT is the first LLM in the ocean domain. (open source)	(Bi et al., 2023)
DeepSeek LLM	DeepSeek-R1	November 29^th^, 2023	January 20^th^, 2025	LLM	DeepSeeke-R1 excels in complex tasks such as mathematics, coding, and natural language reasoning	(Guo et al., 2025a)
Llama 2	Llama 4	July 20^th^, 2023	April 6^th^, 2025	LLM → MLLM	A series of large models released by Meta.	/
Qwen-7B	Qwen2.5-Omni-7B	August 3^rd^, 2023	March 27^th^, 2025	LLM → MLLM	A super large model launched by Alibaba Cloud. (open source)	(Bai et al., 2023)
Kimi Chat	Kimi k1.5	October 10^th^, 2023	January 20^th^, 2025	LLM → MLLM	Kimi k1.5 surpasses GPT-4o by 550% in mathematics, coding, and other capabilities under short-chain thinking mode.	/
Gemma	Gemma 3	February 21^st^, 2024	March 12^th^, 2025	LLM → MLLM	Gemma 3 is a MLLM released by Google. (open source)	(Team et al., 2024, 2025)
PaLM-E	/	March 6^th^, 2023	/	LVM	PaLM-E can integrate vision and language into robot control.	(Driess et al., 2023)
InternImage	InternImage-H	November 10^th^, 2022	October 4^th^, 2023	LVM	A LVM based on deformable convolution. (open source)	(Wang et al., 2023)
PanguCVLM 3.0	PanguCVLM 5.0	July 7^th^, 2023	June 21^th^, 2024	LVM	A LVM that simulates and automates human visual processes.	/
LLaVA	LLaVA-NeXT (Stronger)	April 17^th^, 2023	May 10^th^, 2024	LVM	LLaVA has the ability to align and fuse the visual information of images with the semantic information of text. (open source)	(Liu et al., 2023b)
mPLUG-Owl	mPLUG-Owl3	April 27^th^, 2023	August 20^th^, 2024	LVM → MLLM	mPLUG-Owl is developed by Alibaba DAMO Academy. (open source)	(Ye et al., 2023, 2024)
SPARK 1.0	SPARK 4.0 Turbo	May 6^th^, 2023	October 24^th^, 2024	LVM → MLLM	A new generation of cognitive intelligence model with Chinese as its core.	/
Claude 3	Claude 3.7 Max	March 4^th^, 2024	March 18^th^, 2025	MLLM	A MLLM that primarily focuses on code processing.	/
ERNIE 4.0	ERNIE 4.5	October 17^th^, 2023	March 16^th^, 2025	MLLM	ERNIE is a new generation of Baidu’s large model for knowledge enhancement.	/
ImageBind	/	May 9^th^, 2023	/	MLLM	ImageBind is the first AI model that can bind information from six modes.	(Girdhar et al., 2023)
GPT-4	GPT-4.5	March 14^th^, 2023	February 28^th^, 2025	MLLM	GPT-4.5 significantly enhances its knowledge reserves and emotional intelligence by expanding unsupervised learning and reasoning capabilities.	(Bubeck et al., 2023)
Skywork	Skywork 4.0	April 17^th^, 2023	January 6^th^, 2025	MLLM	Skywork is a series of large models developed by the Kunlun · Skywork team.	(Wei et al., 2023)
Gemini	Gemini 2.5	December 6^th^, 2023	March 26^th^, 2025	MLLM	Gemini is a MLLM launched by Google DeepMind.	(Team et al., 2023)
Sora	/	February 15^th^, 2024	/	MLLM	Sora can create realistic and imaginative scenes from text instructions.	(Peebles and Xie, 2023)
Hunyuan-t1	Hunyuan-t1 (official)	February 17^th^, 2025	March 21^st^, 2025	MLLM	Hunyuan-t1 is a deep-thinking model independently developed by Tencent.	/

The arrow '→' represents the change in the model's category, from its original type to the type of its latest version.

Although LLM and LVM satisfies some functions and takes large models a big step towards artificial general intelligence (AGI), it is not enough to achieve the goal that machines can emulate human thinking and carry out a wide range of general tasks through transfer learning and diverse other modalities without achieving the multimodality of the model (Zhao et al., 2023b). Some large models have implemented multimodality, enabling them to analyze different types of information [GPT-4 (Bubeck et al., 2023), LLaMA, Gemini (Team et al., 2023), ImageBind (Girdhar et al., 2023)] and interact with users. It is worth mentioning that most of the newer large models are MLLMs, and many models that were originally LLMs or LVMs have gradually acquired multimodal capabilities after multiple updates.

However, many current models are generic models and their training datasets are too broad, they cannot provide a satisfactory answer to knowledge in certain professional fields. As Goertzel (2014) believed, for a system to be considered AGI, it is not necessary for it to have infinite generality, adaptability, or flexibility. Therefore, some researchers have optimized and adjusted existing large models and have released some large models specifically for a single field. BloombergGPT can be used in the financial field, showcasing remarkable performance on general LLM benchmarks and surpassing comparable models on financial tasks (Wu et al., 2023c). The meteorological model in panguLM developed by Huawei can provide predictions of variables such as gravity potential, humidity, wind speed, temperature, and pressure within 1 hour to 7 days. Embedding PaLM-E into robots can achieve multiple specific tasks, like visual question answering, sequential robotic manipulation planning, and captioning (Driess et al., 2023). OceanGPT is an expert in various marine science tasks (Bi et al., 2023). It exhibits not only a higher level of knowledge expertise for oceans science tasks but also acquires preliminary embodied intelligence capabilities in ocean engineering. PMC-LLaMA represents the pioneering open-source medical specific language model that demonstrates exceptional performance on diverse medical benchmarks, outperforming ChatGPT and LLaMA-2 with significantly fewer parameters (Wu et al., 2024). The success of large models across diverse fields, as highlighted in this section, underscores their potential to generalize and tackle complex problems, suggesting their applicability to the intricate tasks within agriculture.

### 2.3 Assessing the attention to large models within agriculture

In the past few decades, the advancement of agricultural technology has significantly improved global agricultural production efficiency. According to the forecast released by the food and agriculture organization (FAO) of the United Nations, the global grain production in 2023 was 2.84 billion tons, nearly twice that of the early 20th century. Although global agricultural production efficiency is high, the world population is also constantly growing. Continuously improving agricultural production efficiency is the lifeline of economic development and the foundation for ensuring human food, clothing, and survival needs. Hence, how to make agricultural practices advance is a crucial issue. Next, we will use bibliometric methods in conjunction with practical analysis to explain why large models are important for agriculture.

#### 2.3.1 Bibliometric analysis and data sources

Bibliometrics is a quantitative analysis method that integrates mathematics, statistics, and bibliology, based on mathematical statistics. It focuses on the external characteristics of scientific literature to conduct statistical and visual analyses of the literature (Wang et al., 2019). Keywords encapsulate important information about the research topic. They can intuitively reflect the themes and content of the study, reveal the connections between research contents, results, and characteristics in a particular field, and demonstrate the research dynamics and emerging trends within that area (Li and Zhao, 2015). Our analysis used two methods:

1. Searching for research literature related to large models using the Science Citation Index Expanded (SCI-E) and Social Sciences Citation Index (SSCI) from Web of Science (WoS) with keywords such as “large models”, “large language models”, “large vision models”, or “foundation models”, covering the period from 2019 to 2024.
2. Collecting 3,496 papers from the arXiv in the field of artificial intelligence from 2019 to 2024 and categorizing them by discipline based on keyword searches.

Our analysis of WoS aimed to identify the established trends and peer-reviewed research regarding large models, and specifically, the frequency of agriculture-related keywords within this body of published work. This provides a view of the validated research landscape. Complementarily, we included arXiv to capture the more recent and rapidly evolving trends in artificial intelligence research. arXiv, as a leading platform for pre-prints in AI, offers valuable insights into emerging research directions and the early exploration of applying large models across various disciplines, including potential initial interest in agriculture. Pre-prints often precede formal publication, providing a timelier snapshot of the research frontier.

By analysing both published literature (WoS) and pre-prints (arXiv), we aimed to gain insights from two different perspectives: the established, peer-reviewed research landscape and the more immediate, evolving research front. This allows us to observe both the current state of validated research and the potential emerging trends and initial explorations within the field.

#### 2.3.2 Detailed analysis and design protocol

As described in section 2.3.1, two data sources were used for the specific analysis method: (1) WoS; (2) arXiv. Next, we will elaborate on the details of using these two bibliometric analysis methods.

For the Method 1, after entering the official website of WoS, search in “Web of science Core Collection” and select Science Citation Index Expanded (SCI-EXPANDED) and Social Sciences Citation Index (SSCI) in edition. Both SCI-EXPANDED and SSCI primarily index peer-reviewed journals with established reputations within their respective fields. This ensures a certain level of quality control and scholarly rigor in the literature being analysed. Then search for topics with the keywords “large models”, “large language models”, “large vision models”, or “foundation models”, covering articles from 2019 to 2024, and export the authors, titles, sources and abstracts of these articles in plain text file. Finally, import these plain text files into VOSviever to draw a network of keywords in the field of large models. For the Method 2, we use the keyword “artificial intelligence” to search for articles on arXiv, and crawl the relevant articles from 2019 to 2024, including title, author, abstract and other information, to build a csv file. Then search this file according to relevant keywords. For example, in the medical field, keywords such as medical, healthcare, hospital, etc. are used to filter out relevant articles and count the number. Finally, a graph of the proportion of articles in different fields under the AI ​​domain is constructed.

In this way, we obtained a network map of keyword through the Method 1, and a graph of the proportion of different fields in the AI ​​domain through the Method 2. The specific results and analysis will be explained in the next section.

#### 2.3.3 Analysis results

**Figure 3:**
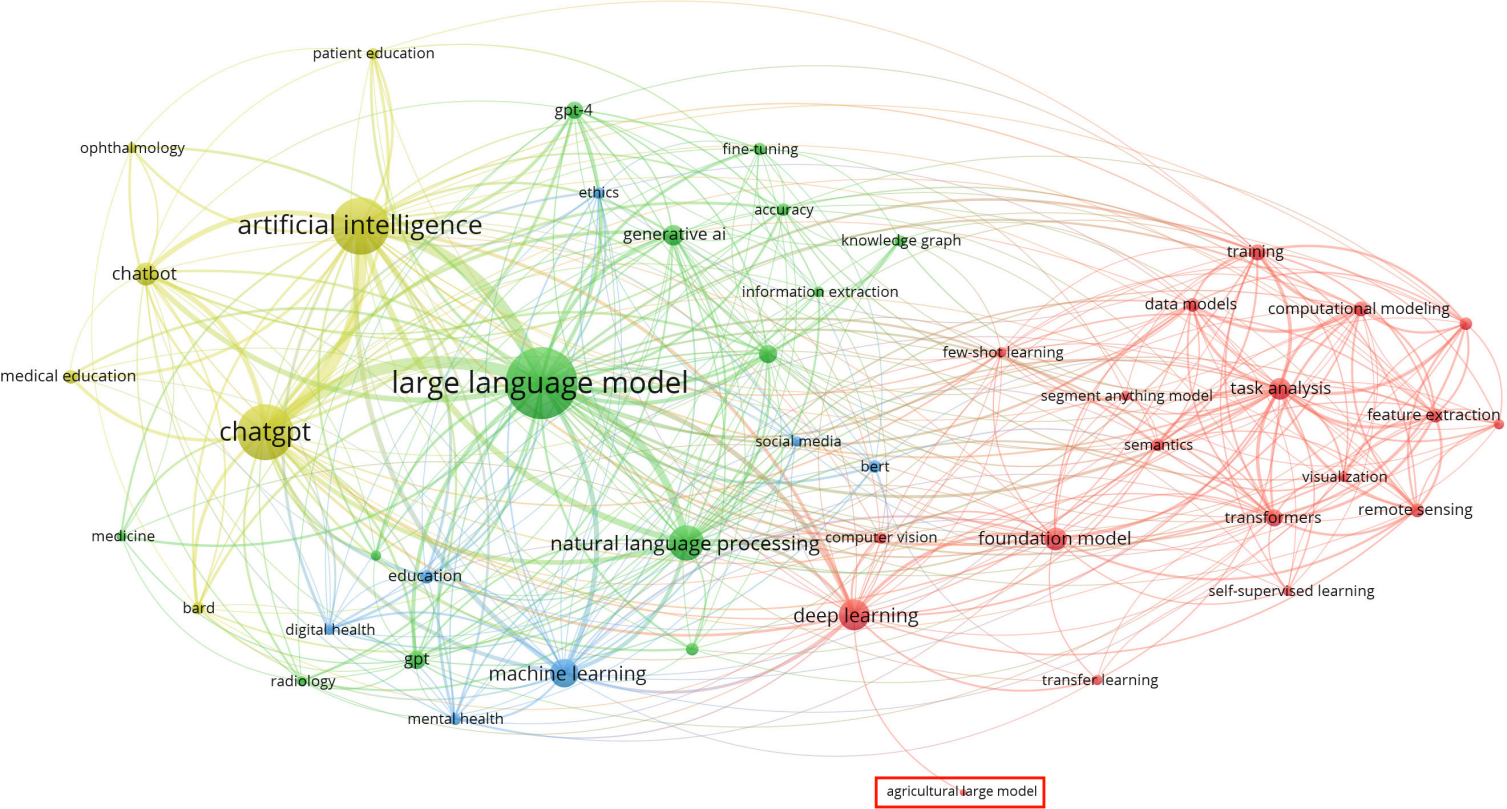
47 keywords co-occurrence network map.

A total of 1,789 papers were filtered using Method 1, and a network diagram of keyword occurrences was generated using VOSviewer. As shown in **Figure 3**, the term ‘agriculture’ appears infrequently in these large model papers, indicating that large models have not received widespread attention in the agricultural field.

The reasons why large models have not received attention in the agricultural sector are diverse. First, large models are a relatively new technology that has emerged in recent years, and many researchers and practitioners in agriculture may not fully understand their capabilities and potential applications in the field. Second, implementing large models often requires substantial computational resources and expertise, which may not be easily accessible in many agricultural environments. Third, agricultural tasks can be very specific and localized, leading people to prefer traditional methods over large models.

**Figure 4:**
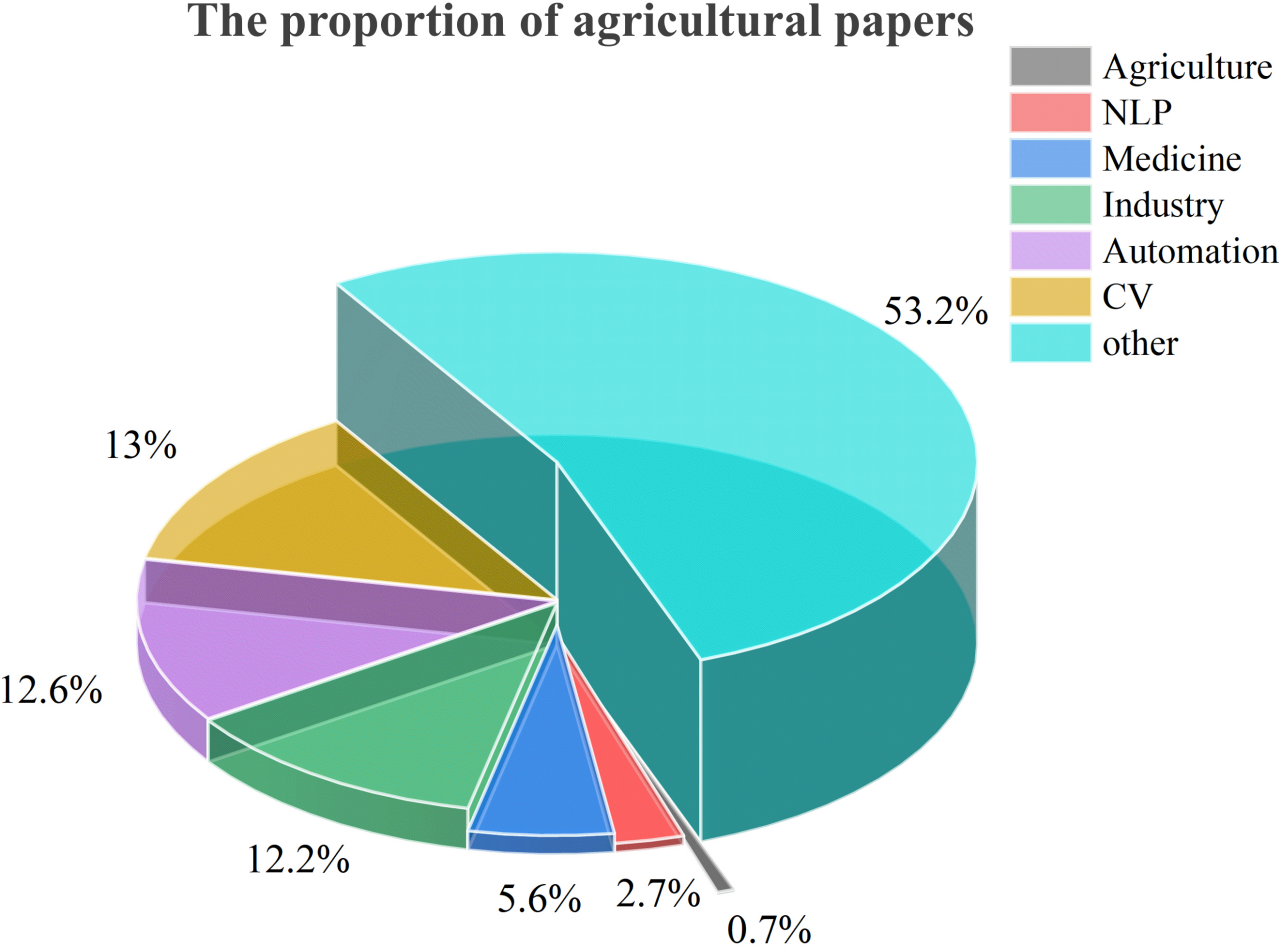
The proportion of arXiv papers on agriculture in AI.

Moreover, **Figure 4** illustrates a difference: the application and research of large models in agriculture are currently limited compared to other fields. This observation from our bibliometric analysis (**Figures 3**, **4**) suggests that despite the evident potential of large models to address agricultural challenges discussed in the introduction, the field is still in the early stages of exploring and adopting this technology. Therefore, a detailed review of their potential applications, associated challenges, and responsible deployment is crucial to guide future research and accelerate their integration into agriculture.

## 3 Large models in agricultural applications

As mentioned in the introduction, agriculture faces multiple challenges, including pests and diseases, seed quality, and crop grading. Large models have demonstrated significant potential in addressing these issues, and some researchers have already developed models specifically tailored for the agricultural domain.

### 3.1 Emerging potential and existing applications of large models in agriculture

#### 3.1.1 Potential and applications

Many large models have emerged, and although they are not yet truly applied in agriculture, their problem-solving capabilities indicate potential prospects in agricultural applications. As shown in **Table 3**, some large models are modifications of existing models, while others are entirely original. For example, given 50 original descriptions related to “wheat rust,” AugGPT can generate 200+ expanded samples covering different growth stages and climatic conditions, thereby enhancing the robustness of disease identification models in complex environments. Aurora is a large model for weather forecasting (Bodnar et al., 2024), and if applied in agriculture, it could enable farmers to schedule activities such as planting, fertilizing, and harvesting based on accurate weather forecasts, as well as proactively mitigate losses from extreme weather events. In addition to ordinary large models, there are also some special existences. HuggingGPT is an AI agent framework designed to orchestrate multiple specialized models, including LLMs like ChatGPT (Shen et al., 2024). It acts as a ‘model coordinator,’ integrating and managing diverse AI components to enhance decision-making in complex scenarios such as agricultural planning. This capability offers possibilities for managing a series of complex agricultural tasks, from planting to harvesting.

**Table 3:** Large models with agricultural potential.

Type	Based	Method	Problem	Application prospect	References
LLM	ChatGPT	GPT-3.5-turbo	Agricultural information extraction	Rapid querying of agricultural information	(Peng et al., 2023)
		AugGPT	Text data augmentation	Few-shot learning for agricultural data	(Dai et al., 2023)
	/	Aurora	Atmospheric prediction	Predicting weather in agriculture	(Bodnar et al., 2024)
LVM	/	MAE, DINO, DINOv2	Plant phenotyping tasks	Monitoring crop health	(Chen et al., 2023a)
	PaLM, ViT	PaLM-E	Robot control	Agricultural intelligent machines or robots	(Driess et al., 2023)
MLLM	SAM	TAM	Object tracking and segmentation in videos	Monitor animals in agricultural farming	(Yang et al., 2023a)

Notably, there are already large models applied in agriculture (**Table 4**). For instance, TimeGPT demonstrates its capability as a smart agriculture tool (Deforce et al., 2024), being used for predicting soil moisture, which helps farmers determine whether the soil is suitable for certain crops. FMFruit showcases the importance of large models in agricultural detection tasks (Li et al., 2024), providing new directions and foundations for the development of robotic harvesting systems. ITLMLP performs disease recognition on cucumbers with limited sample sizes, playing a significant role in agricultural automation and intelligence (Cao et al., 2023).

**Table 4:** Agricultural large models.

Type	Name	Achievement	Significance	References
LLM	TimeGPT	Predicting soil moisture	Contributes to sustainable agricultural practices	(Deforce et al., 2024)
	ChatGPT	Designed a tomato-picking robot	Simplify the design process of agricultural robots	(Stella et al., 2023)
	FMFruit	Identifying multiple types of fruits	Research on robotic harvesting and fruit detection	(Li et al., 2024)
	AgriGPT	Multimodal agricultural knowledge Q&A	Promote precision agriculture practices	(Liu et al., 2023a)
	ShenNong	Development of specialized large models for multiple agricultural domains	Driving agricultural intelligence and comprehensive efficiency improvement	/
	ChatAgri	Cross-linguistic classification of agricultural texts	Provide decision support for precision agriculture	(Zhao et al., 2023a)
LVM	SpectralGPT	Process spectral remote sensing data	Greatly enhanced the processing capability of agricultural spectral data	(Hong et al., 2024)
	SAM	Chicken segmentation and tracking	Facilitates segmentation and tracking tasks in agriculture	(Yang et al., 2023c)
		Agricultural field boundary delineation	Beneficial for PA, crop monitoring, and yield estimation	(Tripathy et al., 2024)
MLLM	ITLMLP	Cucumber disease recognition	Agricultural disease recognition	(Cao et al., 2023)
	AIE-SEG	High-precision segmentation of agricultural imagery	Enables automated field monitoring and yield estimation​	(Xu et al., 2023)

**Tables 2**, **3** demonstrate the feasibility and importance of large models in agriculture, where many agricultural tasks involve complex reasoning. For example, when presented with an image of a soybean field, agricultural scientists or farmers rely on large models to undertake several key steps. Firstly, the large model must identify any abnormal symptoms evident in the soybean leaves, such as leaf wrinkling. Subsequently, it must ascertain the name of the specific problem that troubles plants, such as soybean mosaic. Next, the model needs to determine the underlying cause of the disease, such as soybean mosaic virus. Finally, it must develop an appropriate treatment strategy. This multi-step, cross modal diagnostic and decision-making process is precisely the unique advantage that large models can demonstrate compared to traditional DL models with a single task.

Many question answering (QA) and dialogue systems are designed to address this type of reasoning problem (Rose Mary et al., 2021; Mostaco et al., 2018; Niranjan et al., 2019). For instance, a chatbot based on a RNN is specifically designed to handle questions related to soil testing, plant protection, and nutrient management (Rose Mary et al., 2021). Although, these QA and dialogue systems and chatbots can answer most inquiries without the need for human interaction and with excellent accuracy, they have limited capabilities for complex problems by reason of their small model size as well as of inadequate training data. Therefore, the agricultural domain requires large models to promote the development of QA and dialogue systems and chatbots. The traditional methods for detecting crop pests and diseases mainly rely on special methods such as serology and molecular biology-based technical means, in addition to artificial visual evaluation. Although these methods can accurately determine pests and diseases to a certain extent, they often require a lot of time and money. And some methods of sampling crops often lead to crop damage, which goes against the original intention of diagnosing diseases and pests to protect crops. Therefore, image processing and analysis is an important task for large models in the field of agriculture, and another important task is to embed LVMs into robots to solve some agricultural problems (Weeding, pruning branches, harvesting, etc.) and achieve automated agriculture.

#### 3.1.2 The advantages of agriculture-specific large models

In the field of agriculture, agriculture-specific models can offer notable advantages over general large models, particularly by effectively integrating diverse, domain-relevant data modalities such as image, text, and crucial label information. This multimodal strategy, often employing techniques like combined contrastive learning methods within a unified feature space, allows these models to address the prevalent challenge of data scarcity in agriculture more effectively than models relying solely on single modalities or vast, generic pre-training datasets.

By explicitly learning and leveraging the semantic correlations between visual features (e.g., specific crop disease symptoms) and related textual descriptions or categorical labels, agriculture-specific models can develop more comprehensive, robust, and discriminative representations tailored to the nuances of the field. For example, ChatAgri excels in the specific task of agricultural visual diagnostics (Zhao et al., 2023a). A general MLLM might identify visual anomalies, and lack the specialized knowledge to accurately name the specific agricultural disease or pest, understand its lifecycle, or recommend appropriate, targeted treatments. Especially when faced with limited training samples, agriculture-specific large models may perform better compared to models with poor adaptability. Unlike general large models that often require vast datasets for pre-training and may not adapt well to fine-tuning on limited agricultural data, ITLMLP is designed to be effective with small sample sizes. It extracts richer and more discriminative features from scarce data, leading to significantly higher recognition accuracy (achieving 94.84% in their paper) compared to general large models to the same small dataset (Cao et al., 2023).

Furthermore, their focused training enables them to better identify and weigh agriculturally significant features, accurately discerning subtle but critical patterns for tasks like disease recognition while potentially mitigating the influence of irrelevant background elements, ultimately resulting in improved accuracy, reliability, and greater practical applicability within the complex agricultural environment.

### 3.2 Leveraging large language models for agricultural data processing, insights, and decision support

LLM can play many roles in the agricultural domain, such as processing and generating agricultural data, providing insights into agricultural production work, and supporting agricultural decision-making for farmers.

#### 3.2.1 Large language models for processing and generating agricultural data

##### 3.2.1.1 Information extraction

LLMs can extract structured information from unstructured agricultural text data. First, the text is divided into individual tokens and LLMs represent each token as a numerical vector called a word embedding. Then, LLMs analyse the surrounding context of each token to understand its meaning within the sentence or document, and identify and categorize named entities within the text, like names of individuals, locations, organizations, or specific agricultural terms. Finally, LLMs employ techniques like information extraction to identify and extract structured information from unstructured text (Involve identifying relationships between entities, extracting key facts, or populating knowledge graphs). LLMs extract information from data using a process known as NLP. Beyond mere extraction, modern LLM applications increasingly employ a paradigm known as retrieval-augmented generation (RAG). In this approach, the LLM first retrieves relevant, up-to-date information from external, domain-specific knowledge bases—such as recent agronomic research, real-time market prices, or local pest outbreak databases. This retrieved context then “augments” the model’s input, enabling it to generate responses that are not only more accurate and timelier but also grounded in verifiable sources, thereby significantly mitigating the risks of data lag and factual inaccuracies in the agricultural domain (Gao et al., 2023).

##### 3.2.1.2 Agricultural data generation

Generative AI models are a multimodal LLM, which is the MLLM mentioned above. An obstacle encountered when applying specialized CV algorithms to agricultural vision data is the insufficient availability of training data and labels (Qi et al., 2017; He et al., 2017). In addition, collecting data that encompasses the wide range of variations caused by season and weather changes is exceedingly challenging. Acquiring high-quality data requires a lot of time, and labelling them is even more costly (Zhou et al., 2017). To address these challenges, one approach is to fine-tune multimodal generative LLMs on the target agricultural data domain. This allows the models to generate massive training data and labels, thereby constructing an augmented training set that closely resembles the distribution of the original data (Dai et al., 2023). Besides, text-based generation models can generate images (Rombach et al., 2022) and videos (Ho et al., 2022) of specific scenes based on text descriptions, thereby supplementing training datasets that may lack certain visual content. This helps in expanding the training data and improving the performance of downstream models.

#### 3.2.2 Large language models provide insights

LLMs possess the capability to analyse textual data and uncover trends in agricultural practices, market conditions, consumer preferences, and policy developments. Through analysis of agricultural text data from sources such as news articles, reports, and social media, these models can offer valuable insights into market dynamics and pricing trends (Yang et al., 2024). This provides support for farmers to understand domains outside of agriculture. Many researchers believe that the integration of LLMs into different stages of designment and development for agricultural applications is also experiencing a noticeable rise (Stella et al., 2023; Lu et al., 2023). In (Stella et al., 2023) study, Stella et al. incorporated LLM into the design phase of robotic systems. They specifically focused on designing an optimized robotic gripper for tomato picking and outlined the step-by-step process. In the initial ideation phase, they leveraged LLMs like ChatGPT (Bubeck et al., 2023) to gain insights into the possible challenges and opportunities associated with the task. Building upon this knowledge, they identified the most promising and captivating pathways, engaging in ongoing discussions with the LLM to refine and narrow down the design possibilities. Throughout this process, the human collaborator harnesses the expansive knowledge of the LLM to tap into insights transcend their individual expertise. In the following stage of the design process, which emphasizes technical aspects, the broad directions derived from the collaboration need to be transformed into a real, completely functional robot. Although LLMs do not provide comprehensive technical support, they can offer their own insights on whether the technology is feasible, helping researchers reduce the risk of failure.

Presently, LLMs lack the ability to generate comprehensive CAD models, evaluate code, or independently fabricate robots. Nevertheless, advancements in LLM research suggest that these algorithms can offer significant assistance in executing software (Chen et al., 2021), mathematical reasoning (Das et al., 2024), and even in the generation of shapes (Ramesh et al., 2022). Lu et al. specifically focused on the utilization of LLMs for organizing unstructured metadata, facilitating the conversion of metadata between different formats, and discovering potential errors in the data collection process (Lu et al., 2023). They also envisioned the next generation of LLMs as remarkably potent tools for data visualization (Bubeck et al., 2023), and anticipated that these advanced models will provide invaluable support to researchers, enabling them to extract meaningful insights from extensive volumes of phenotypic data.

Although LLMs provide insights can indirectly help farmers solve a small number of agricultural tasks, it’s important to note that their insights should be used in conjunction with human judgment and domain expertise. That is to say, the insights provided by LLMs cannot be separated from human experience.

#### 3.2.3 Large language models empower decision-making for farmers

According to a recent study, ChatGPT demonstrates the ability to comprehend natural language requests, extract valuable textual and visual information, select appropriate language and vision tasks, and effectively communicate the results to humans (Shen et al., 2024). Shen et al. proposed a system named HuggingGPT to solve AI tasks. HuggingGPT is a collaborative AI task resolution framework built on LLMs. This system connects LLM with AI models through language interface, and these AI models are derived from HuggingFace. This coordinating capability positions LLMs as the core of modern AI Agents. As the core of decision-making, LLM can be applied to agriculture to help solve the tasks proposed by farmers (Shen et al., 2024).

**Figure 5:**
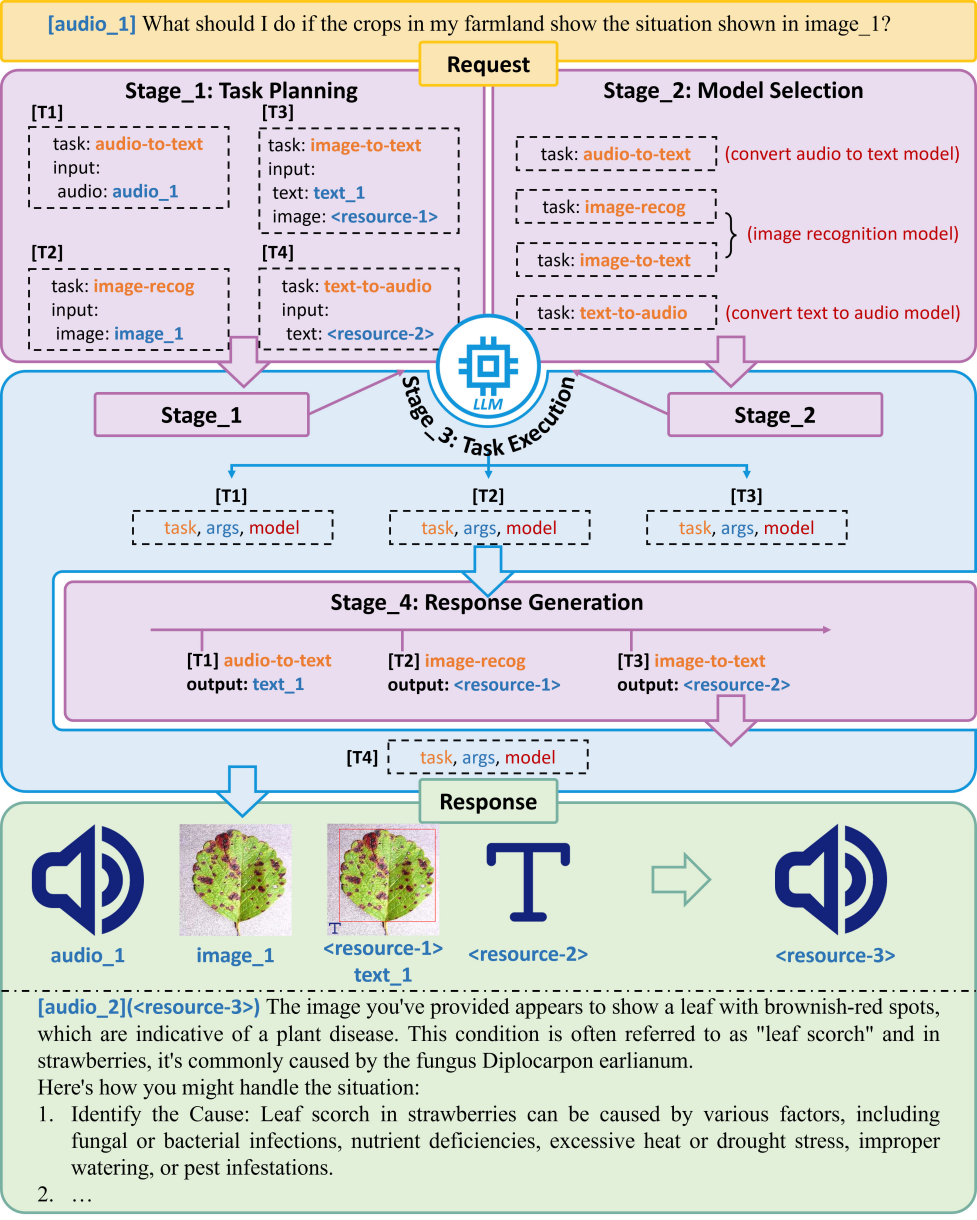
An LLM-based AI Agent architecture for agricultural decision-making support.

An AI Agent is an autonomous system that perceives its environment, reasons, plans, and acts to achieve specific goals. As shown in **Figure 5**, the LLM acts as the agent’s “brain”, performing crucial functions. When receiving a task request, LLM first divides the total task into subtasks and selects the appropriate AI model based on the needs of each subtask. For example, converting farmers’ audio into text requires the use of an audio to text model [Amazon transcribe, Whisper (Radford et al., 2023)]; It is also necessary to recognize the sent image and integrate the text obtained from the audio conversion in the previous step to obtain a text-response (vit-gpt2); Considering that some farmers may have had limited access to formal education, it is necessary to further convert text-response into audio and ultimately obtain the audio-response [Fastspeech (Ren et al., 2019, 2020)]. Although LLM does not play a role in solving problems throughout the entire system, as a “conductor”, it can coordinate various AI models to complete subtasks, thereby gradually solving complex tasks and playing a core role in decision-making support.

### 3.3 The role of large vision model in image processing, analysis, and agricultural automation

While LLMs excel in processing textual and knowledge-based information, many agricultural tasks fundamentally rely on visual data. Using a LVM to judge crop related information can not only greatly improve the time required for judgment, but also indirectly reduce the damage caused to crops. Moreover, after crops are invaded by pests and diseases, their color, texture, spectral characteristics will undergo certain changes, all of which are related to CV.

#### 3.3.1 Image processing and analysis

At present, there are four types of methods for obtaining crop image information: 1) ordinary channels, taking photos to obtain images; 2) obtaining remote sensing images through agricultural machinery near the ground; 3) obtaining remote sensing images through aircraft monitoring platforms (Yuan et al., 2022); 4) obtaining remote sensing images through satellites (Zhang et al., 2019). Remote sensing can provide large-scale land use and land cover information. By analysing satellite images or high-altitude images, various surface information can be identified, such as surface conditions, soil moisture, vegetation coverage, and crop growth status (Khanal et al., 2020). Classifying and segmenting from limited examples obtained from remote sensing is a significant challenge. Regarding this, Wu et al. (2023b) put forward GenCo (a generator-based two-stage approach) for few-shot classification and segmentation on remote sensing and earth observation data. Their approach presents an alternative solution for addressing the labelling challenges encountered in the domains of remote sensing and agriculture. Spectral data can provide rich insights into the composition of observed objects and materials, especially in remote sensing applications. The challenges faced in processing spectral data in agriculture include: 1) effectively processing and utilizing vast amounts of remote sensing spectral big data derived from various sources; 2) deriving significant knowledge representations from intricate spatial-spectral mixed information; 3) addressing the spectral degradation in the modelling of neighbouring spectral relevance. Hong et al.’s SpectralGPT empowers intelligent processing of spectral remote sensing big data, and this LVM has also demonstrated its excellent spectral reconstruction capabilities in agriculture (Hong et al., 2024). Due to multispectral imaging (MSI) and hyperspectral imaging (HSI) make it possible to monitor crop health in the field. The integration of remotely sensed multisource data, such as HSI and LiDAR (Light detection and ranging), enables the monitoring of changes occurring in different parts of a plant (Omia et al., 2023). By using a large visual model to analyse these spectral data, the obtained crop health information can help farmers quickly and accurately identify diseases and treat them, reducing the loss of crop yield.

**Figure 6:**
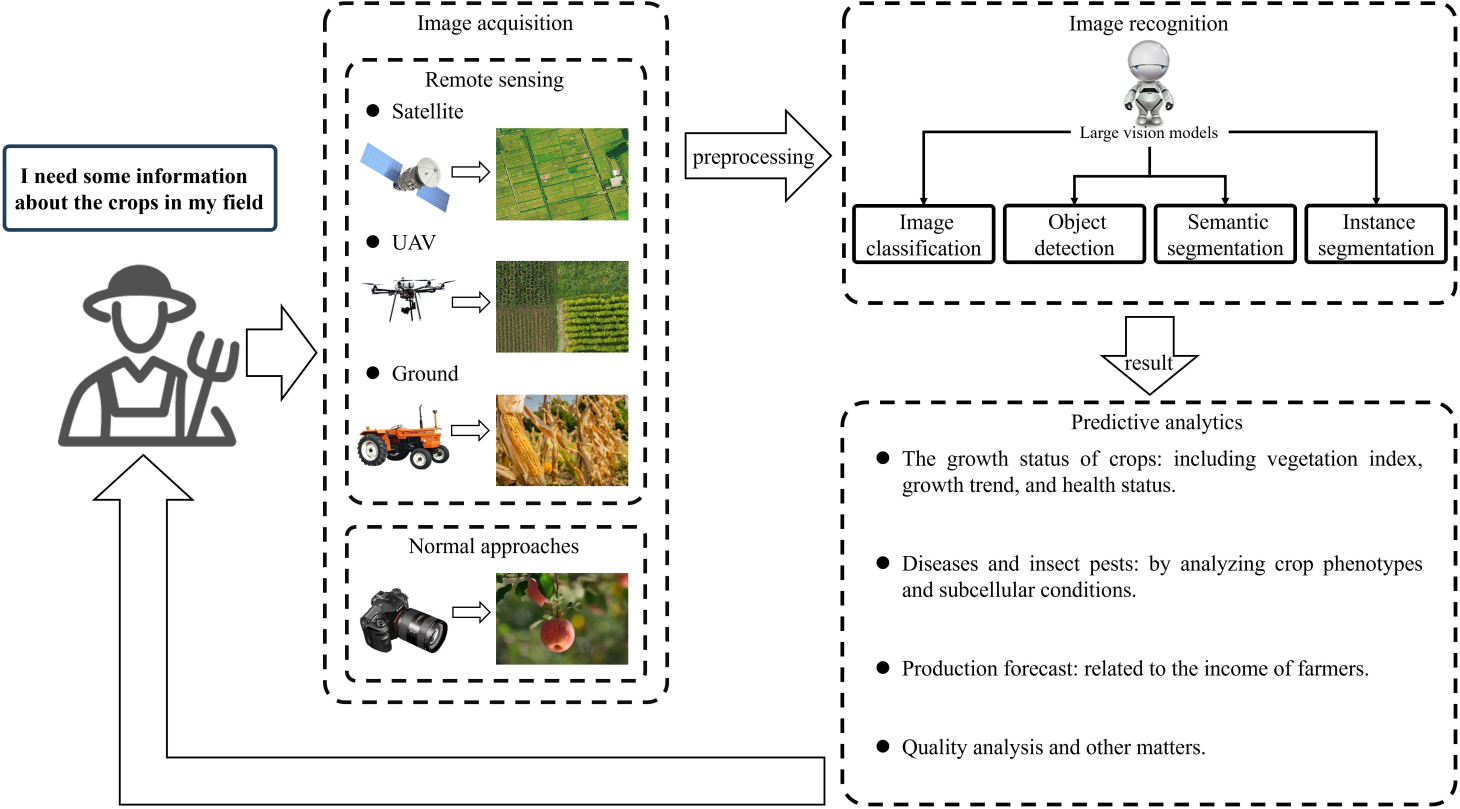
Farmers can obtain crop information through the process of image acquisition, image recognition, predictive analytics.

Studies suggest that the use of LVMs for image recognition and predictive analysis of crop information is often more effective than traditional ML algorithms. When farmers need to obtain crop information, four types of image acquisition methods can be used to obtain crop image information (**Figure 6**). Then, the image information is processed through image recognition (Divided into four tasks: image classification, object detection, semantic segmentation, instance segmentation), and the identified results need to be further predictive analytics to obtain crop information that farmers can understand.

In addition to obtaining information by analysing the phenotypic characteristics of crops, Feng et al. (2022) developed a traditional DL model called organelle segmentation network (OrgSegNet). OrgSegNet is capable of accurately capturing the actual sizes of chloroplasts, mitochondria, nuclei, and vacuoles within plant cell, further inspecting plant phenotypic at the subcellular level. They have tested two applications: 1) A thermo-sensitive rice albino leaf mutant was cultivated at cold temperature conditions. In the transmission electron microscope images (TEMs), the albinotic leaves lacked typical chloroplasts, and OrgSegNet failed to identify any chloroplast structures; 2) Young leaf chlorosis 1 (Ylc1). Young leaves of the ylc1 mutant showed lower levels of chlorophyll and lutein compared to corresponding wild type, and its TEM analysis further revealed a noticeable loose arrangement of the thylakoid lamellar structures. It can be imagined that if a large model is used to replace DL algorithms, the recognition of subcellular cells may perform better, and the recognition results can be further predictive analytics to obtain information that non plant experts can also understand.

#### 3.3.2 Automation and robotics

Enhancing the intelligence of agricultural robots is a crucial application area for large models. Conventional agricultural robot systems, typically composed of perception, decision-making, and actuation modules, often struggle with complex visual perception and intelligent, real-time decision-making, especially in unstructured and dynamic farm environments (Yang et al., 2023b; Hamuda et al., 2016). Integrating large models is a promising approach to overcome these limitations and significantly enhance the intellectual features of agricultural robots.

Current LVMs can be used in drones to monitor crops and obtain information on their growth, disease, yield, and other factors (Ganeshkumar et al., 2023; Chin et al., 2023; Pazhanivelan et al., 2023). In addition to the above functions, ground machines that used LVMs can also be used for harvesting and classifying crops, as well as detecting pests up close. In (Yang et al., 2023c), a LVM, segment anything model (SAM) (Kirillov et al., 2023), uses infrared thermal images for chicken segmentation tasks in a zero-shot means. SAM can be used in agriculture to segment immature fruits on a fruit tree and quickly achieve yield prediction. Yang et al. (2023a) subsequently proposed the Track Anything Model (TAM) by combining SAM and video. Unfortunately, TAM places more emphasis on maintaining short-term memory rather than long-term memory. Nevertheless, based on its capabilities, TAM still has great potential in the agricultural field. If its long-term memory ability can be improved, it can monitor early changes in crop diseases and provide early warning to farmers. Embedding LVMs such as SAM and TAM into robots can not only achieve automation in agriculture, but these LVMs themselves can help achieve automation in agricultural robot design.

Beyond perception, large models are also revolutionizing the design process of agricultural robots. As mentioned previously, Stella et al. (2023) demonstrated using LLMs like ChatGPT to assist in designing an optimized robotic gripper for tomato picking. With the latest multimodal versions like GPT-4.5, designers can now input not only text descriptions but also sketches to partially automate the robot design process. This integration of LVMs for perception and LLMs for both control logic and design automation marks a significant step towards fully autonomous agricultural systems.

### 3.4 Integration of multimodal models

**Figure 7:**
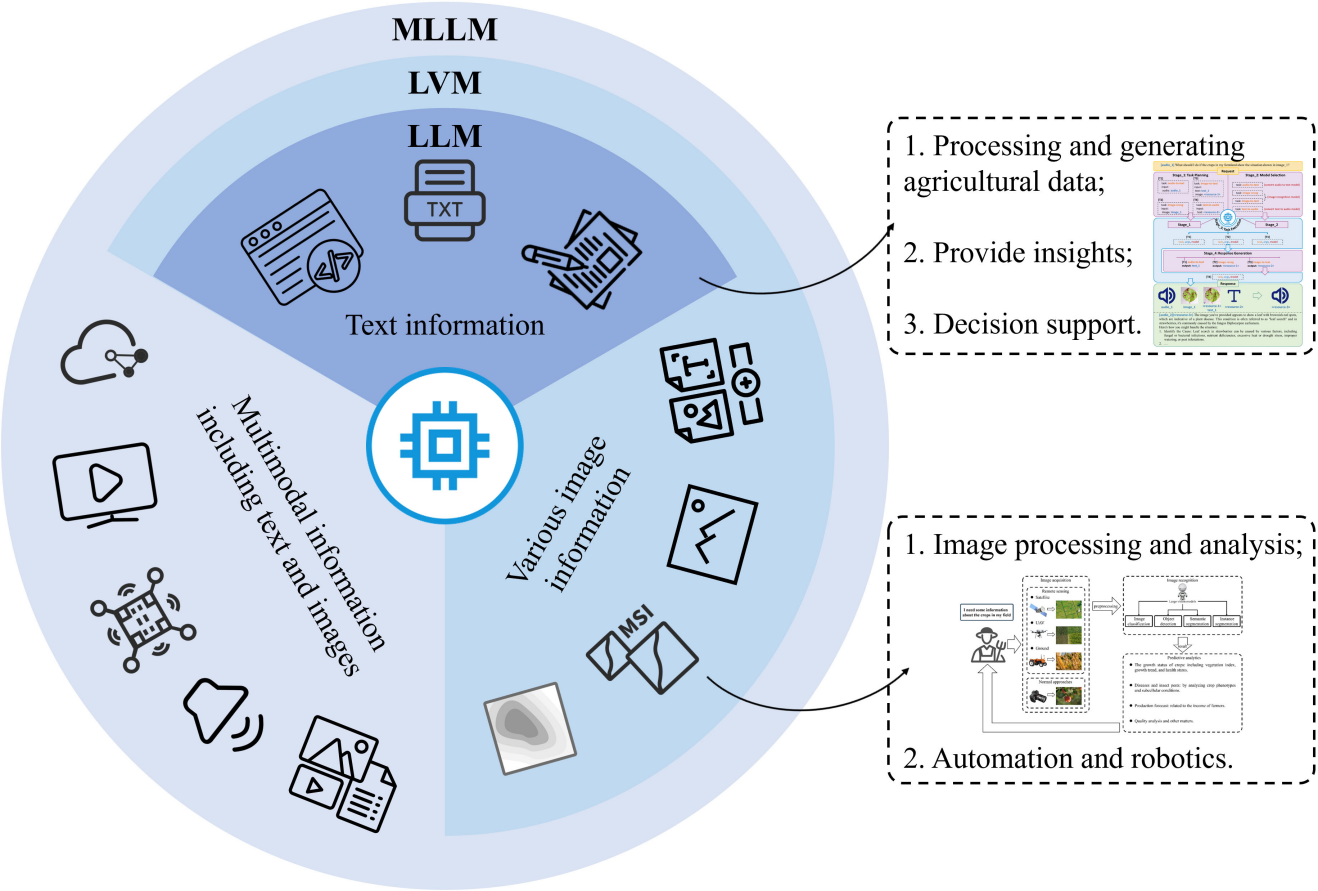
Multimodal information fusion analysis driven by MLLM.

LVMs provide powerful capabilities for visual analysis and robotic perception. However, the most complex agricultural challenges often require integrating information from multiple sources. MLLM recently has emerged as a prominent research hotspot (**Figure 7**), which uses powerful LLMs as a core to tackle multimodal tasks (Yin et al., 2023). In recent years, many researchers have utilized and merged diverse types of data inputs, such as text, images, audio, video (Zhang et al., 2023a), sensor data (Driess et al., 2023), depth information, point cloud (Chen et al., 2024), and more.

The agricultural community has started exploring the realm of multimodal learning in agricultural applications. By incorporating multimodal learning techniques, the agricultural community seeks to unlock new opportunities for optimizing various agricultural processes and achieving improved outcomes. As an example, Bender et al. have released an open-source multimodal dataset specifically curated for agricultural robotics (Bender et al., 2020). This dataset was collected from cauliflower and broccoli fields and aims to foster research endeavors in robotics and ML within the agricultural domain. It encompasses a diverse range of data types, including stereo color, thermal, hyperspectral imagery, as well as essential environmental information such as weather conditions and soil conditions. The availability of this comprehensive dataset uses as a precious resource for advancing the development of innovative solutions in agricultural robotics and ML. Cao et al. (2023) proposed a novel approach for cucumber disease recognition using a MLLM that incorporates image-text-label information. Their methodology effectively integrated label information from many domains by employing image-text multimodal contrastive learning and image self-supervised contrastive learning. The approach facilitated the measurement of sample distances within the common image-text-label space. The results of the experiment demonstrated the effectiveness of this innovative approach, achieving a recognition accuracy rate of 94.84% on a moderately sized multimodal cucumber disease dataset.

Nevertheless, it is important to highlight that existing models primarily rely on text-image data and are mostly limited to QA tasks. There is a noticeable lack of applications in the realm of agricultural robotics that incorporate inputs like images, text, voice (Human instructions), and depth information (From LiDAR or laser sensors). These agricultural robots, commonly deployed for tasks such as fruit picking or crop monitoring (Tao and Zhou, 2017), present a significant opportunity for the integration of multimodal data sources to enhance their capabilities. In short, large models lacking a high degree of multimodality perform fewer tasks and lack good applicability.

### 3.5 The choice between large models and traditional models

The decision to implement either a large model or a traditional model in agriculture is not straightforward. It involves considering a multitude of factors, such as the volume and quality of available data, the required model generalizability, and the practical limits on computational power and inference speed. However, by analyzing the studies of Deforce et al. (2024); Zhao et al. (2023a), and Cao et al. (2023), we found that these considerations can be effectively categorized under two primary factors: Data and deployment conditions. Similar to how large models are divided into LLM, LVM, and MLLM, traditional models can be classified according to the specific agricultural task, falling into the categories of NLP, CV, and multimodal. For instance, models like AGRONER and PSO-LSTM are designed to handle NLP tasks (Veena et al., 2023; Zheng and Li, 2023), AG-YOLO and CMTNet address CV tasks (Lin et al., 2024; Guo et al., 2025b), while ITK-Net and Multi-ResNet34 are tailored for multimodal applications (Zhou et al., 2021; Zhang et al., 2022a). Before selecting a model, it is best to first determine which category the agricultural task belongs to.

**Figure 8:**
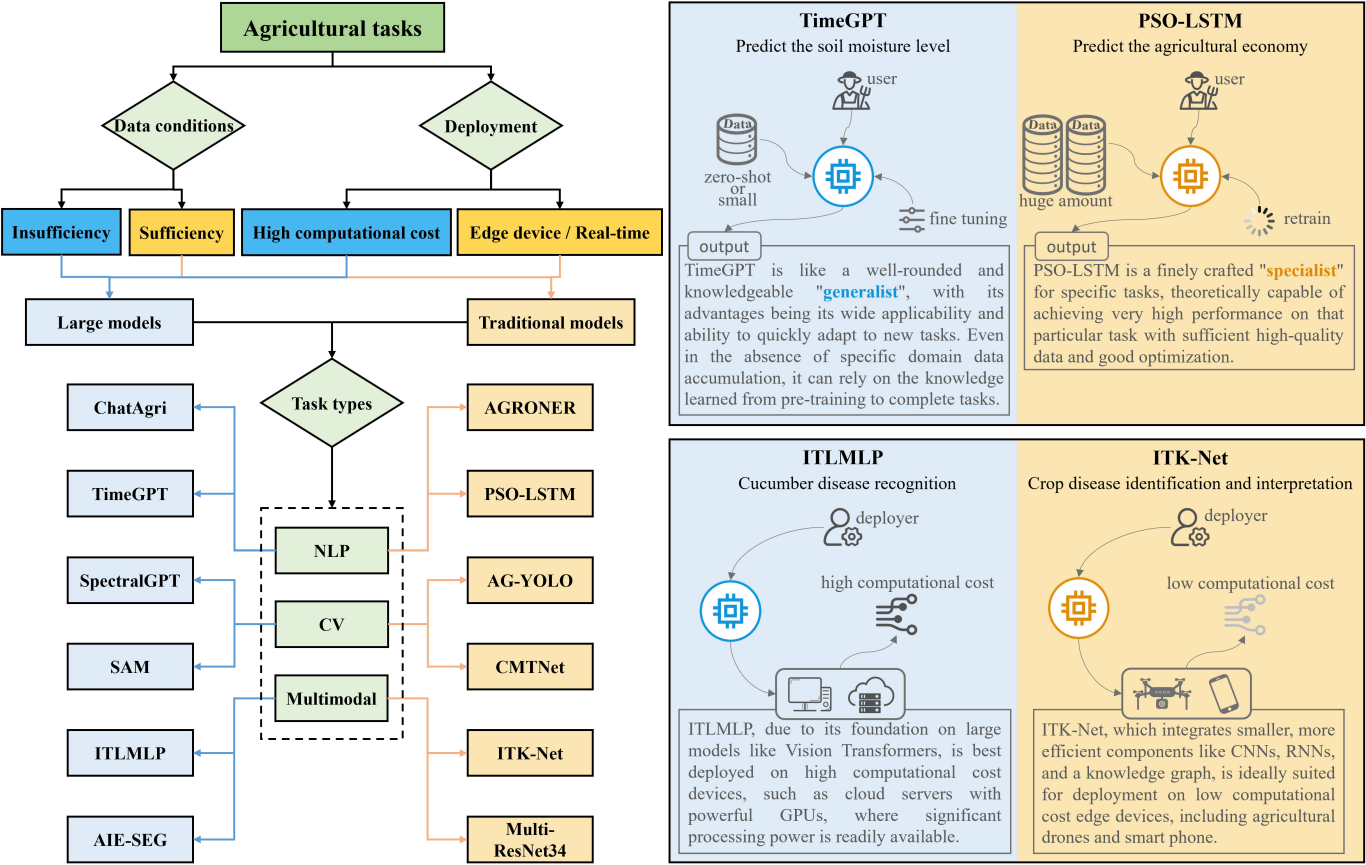
Comparison of large models and traditional models for agricultural tasks.

When approaching an agricultural task, a critical step is to assess the sufficiency of available data. If a substantial volume of high-quality, task-specific data is available, a traditional model becomes a good option. Conversely, in scenarios marked by data insufficiency, leveraging a large model is often the more suitable choice. **Figure 8** presents a comparative analysis of traditional models versus large models based on data conditions and deployment constraints. PSO-LSTM can be retrained on abundant data, and it can deliver superior performance for a particular agricultural task, thus positioning this model as a “specialist”. TimeGPT, on the other hand, functions as a “generalist”, capable of handling diverse, non-specific agricultural tasks using only minimal fine-tuning or a zero-shot approach in data-scarce situations, thereby avoiding the need for complete model retraining for each new task (Deforce et al., 2024). The pre-embedded knowledge within large models can effectively compensate for the lack of domain-specific data.

On the other hand, deployment conditions are also a crucial factor in model selection. While devices with high computational capacity can deploy both large models and traditional models, the significant computational and time costs associated with large models make them unsuitable for edge devices and systems requiring real-time response. For an agricultural task that requires the model to be deployed on an edge device with real-time detection needs, ITK-Net is the pragmatic and superior choice due to its efficiency and low resource requirements (Zhou et al., 2021). While the ITLMLP model proposed by Cao et al. (2023) also targets crop disease recognition, it is suited for deployment only on devices that can handle high computational costs. As a large model, ITLMLP’s deployment conditions are considerably more stringent than those of ITK-Net, the traditional model. However, this does not imply that ITLMLP is without its merits. Its value lies not in real-time field deployment, but in its powerful offline analysis capabilities. By batch-processing vast agricultural data stored on cloud servers, it can perform in-depth retrospective diagnostics and trend analysis. Leveraging its powerful feature extraction and generalization capabilities, acquired from pre-training on large-scale data, ITLMLP can conduct reclassification of historical disease data, compile statistics on disease occurrence frequencies across different periods, and uncover potential correlations between image features and specific environmental descriptions. By the way, by optimizing the model architecture, using efficient inference algorithms, and utilizing hardware acceleration techniques, the real-time performance of LVMs can be improved to a certain extent (Chen et al., 2023b). In addition, we have also discovered that ITLMLP could process a large dataset to generate highly accurate annotated labels, which can then be used to train smaller, more efficient models like ITK-Net. This creates a synergistic ecosystem where the power of large models enables the effectiveness of traditional models on the edge.

The choice between a large model and a traditional model for agricultural tasks is not a matter of one being definitively superior to the other, but rather a strategic decision based on a careful evaluation of trade-offs. Large models, with their powerful generalization capabilities, offer a robust solution for data-scarce environments, while traditional models excel in data-rich scenarios where their specialized nature can be fully leveraged. Similarly, the high computational cost of large models makes them suitable for offline, server-based analysis, whereas the efficiency of traditional models is indispensable for real-time, on-device deployment.

## 4 Ethical issues and responsible use of large vision and language models in agriculture

As large models demonstrate their powerful potential and are increasingly applied to agricultural tasks (referencing section 3), it is crucial to critically examine the ethical and societal implications of their deployment. However, there are often ethical and responsibility issues in the development and deployment process of AI today. The digital gap between those who have the resources to develop and utilize large models and those who cannot afford to do so creates an inequality in accessing large models, resulting in an unfair distribution of risks and benefits (Harfouche et al., 2024). Not only that, this divide can be exacerbated by the presence of AI biases (Dara et al., 2022; Ryan, 2023). Accordingly, to ensure ethical issues and responsible use of large models, this chapter starts from the ethical and responsibility issues in the agricultural large models and explore corresponding measures.

### 4.1 Ethical considerations in the deployment of large models in agriculture

Predicting and solving ethics problems of large models in agriculture is a critical scientific and societal challenge. Although large models point the way for the future of smart agriculture, due to their characteristic of being influenced by close association, large models often learn some bad knowledge in addition to useful knowledge. Ethical issues have always been an indispensable topic of discussion in the process of technological progress (Such as the ethical issues discussed by Holmes et al. in the field of education regarding educational AI (Holmes et al., 2022)), and we also need to pay attention to ethics issues when using large models in the agricultural direction. As mentioned below, many relevant institutions and personnel have put forward their own ideas on ethical issues related to large models.

**Figure 9:**
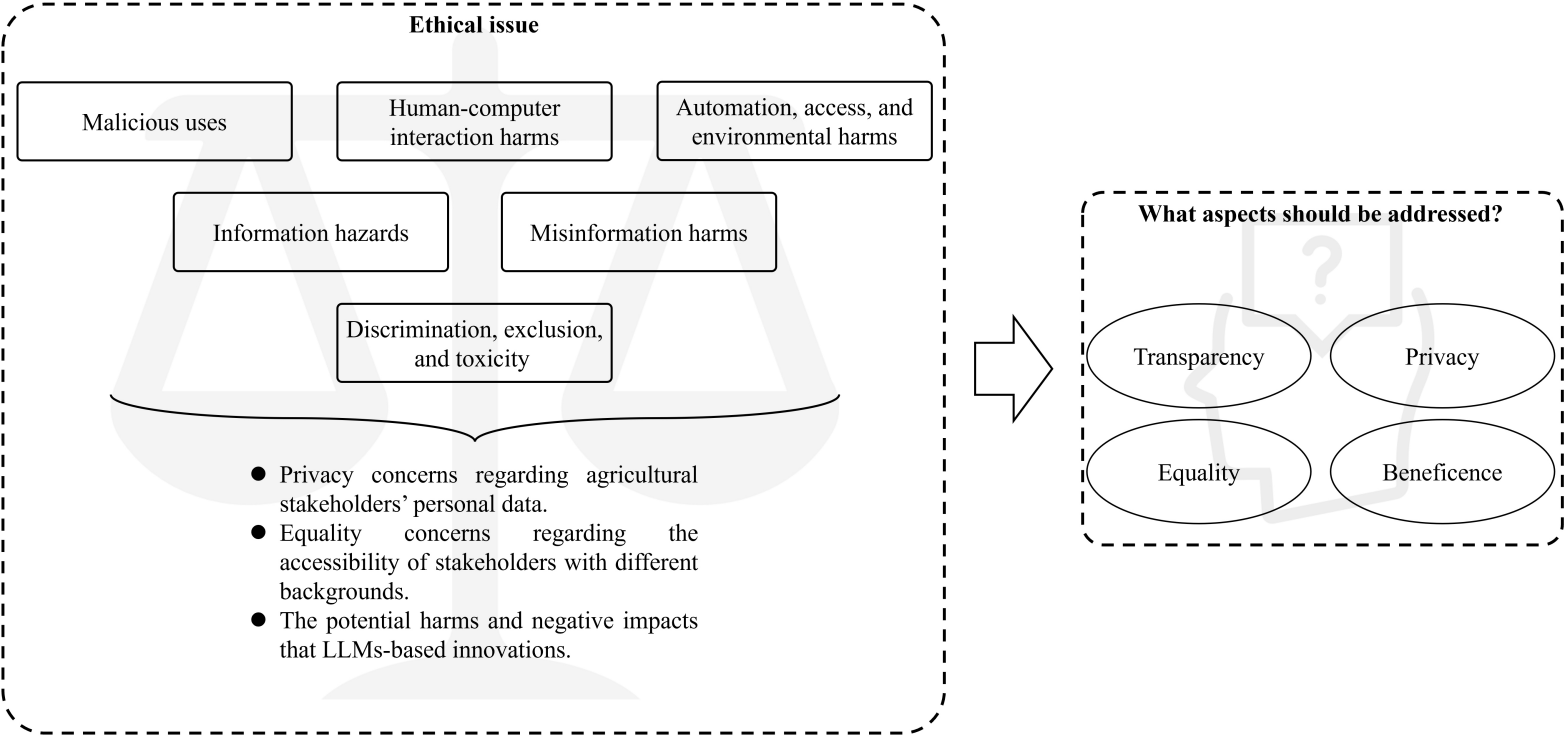
The ethical issues faced by large models.

Weidinger et al. (2021) put forward six types of ethical risks (**Figure 9**): 1) Malicious uses, 2) Human-computer interaction harms, 3) Automation, access, and environmental harms, 4) Information hazards, 5) Misinformation harms, and 6) Discrimination, exclusion, and toxicity. Understanding these issues can help us responsibly use large models in the agricultural field.

1. Malicious uses. Prior to the release of GPT-4, OpenAI hired a team of 50 experts and scholars to conduct a six-month adversarial test on GPT-4. Andrew White, a professor of chemical engineering at the University of Rochester who was invited to participate in the test, stated that early versions of GPT-4 could assist in the manufacture of chemical weapons and even provide a convenient manufacturing location. From the perspective of the agricultural sector, if this issue is not properly addressed, some may use large models to learn ways to destroy other people’s farmland for the sake of profit, thereby allowing themselves to have a larger market. Over time, this will lead to vicious competition in the market.
2. Human-computer interaction harms. The potential harms of human-computer interaction arise when users excessively trust a large model or mistakenly treat it as human.
3. Automation, access, and environmental harms. The large model can give rise to automation, access, and environmental harms due to its potential environmental or downstream economic impacts.
4. Information hazards. Due to the involvement of information from different countries, religions, and ethnicities, model outputs leaking or inferring sensitive information often led to political violence.
5. Misinformation harms. A study discussed the potential risks of using poorly performing large models. The original intention of this study was to provide a natural language generation model in MOOC to respond to students and improve their participation rate (Li and Xing, 2021). Even so, due to the poor performance of the model, the corresponding negative results further reduced the enthusiasm of students. If a poorly performing large model is used in the agricultural field, it may mislead farmers in their judgment (Such as analysing incorrect disease types), not only causing further damage to crops in the farmland, but also making farmers increasingly distrust the large model. For this phenomenon, Angelone et al. proposed that warning labels can be applied to the content generated by the large model (Angelone et al., 2022), but this also involves the trust issue of the large model in its own generated results.
6. Discrimination, exclusion, and toxicity. Two researches have indicated that potential discrimination, exclusion, and toxicity issues may occur if adopting a model that is accurate but unfair (Sha et al., 2021; Merine and Purkayastha, 2022).

Despite Weidinger et al.’s viewpoint can provide us with a fundamental understanding of the risks associated with large models, manners of systematic ethical supervision of large models’ research and innovation (R&I) are especially restricted. Coincidentally, the European Commission has officially approved comprehensive “ethics guidelines for trustworthy AI” specifically designed for R&I. These guidelines require that principal investigators recognize and tackle the ethical matters raised by their proposed research. Principal investigators are also required to adhere to ethical principles and relevant legislation in their work. In a similar vein, Stanford University’s Ethics and Society Review necessitates researchers to distinguish potential societal hazards associated with their research and incorporate mitigation measures into their research design (Bernstein et al., 2021).

Furthermore, projects with large models have a vast amount of data and often raise ethics issues. For instance, while raw plant science data itself may not inherently fall within the scope of the European Union General Data Protection Regulation (GDPR) as personal data, it can become subject to GDPR regulations when linked to identifiable individuals or specific farm locations tied to individuals, creating complex challenges concerning data ownership and privacy protection (Harfouche et al., 2024). Thus, relevant guidelines must consider code of conduct for data sharing, privacy protection, and the overall governance of datasets.

### 4.2 Responsible use in agriculture

With the expanding development and utilization of large models, there is a growing recognition of the need for agile and effective regulatory oversight. To address this issue, it may be necessary to use AI technology to assist in overseeing the development and deployment of large models. Regarding this aspect, the AI Act, which has been jointly agreed upon by the European Parliament and the Council of Europe, represents the first comprehensive set of harmonized rules on a global scale. It promotes responsible large model designment and development by regulating large model across various applications and contexts based on a risk-based framework. Within the framework, careful consideration must be given to the level of risk involved and how to evaluate different large models as risk-free or low-risk.

To evaluate the risk level of a large model, we focus on four aspects: transparency, privacy, equality, and beneficence. On the other hand, in addition to developing and adhere to a strong regulatory framework that guides the development, deployment, and use of large models, regulatory methods also need to be considered. Consider the potential societal impact, potential harms, and long-term implications of the technology. Firstly, due to the wide applicability of large models, we cannot make a one size fits all approach. Regulation must adapt to specific issues in different domains. The United States’ food and drug administration (FDA) has tailored potential regulatory methods for AI and ML technologies used in medical devices, categorizing them into three major categories based on risk levels: Class I (Low risk), Class II (Moderate risk), and Class III (High risk). Large models in agriculture can also be regulated according to the FDA’s approach, dividing them into several types of models ranging from low risk to high risk. For example, genetically modified crops may have environmental impacts, food safety issues, and ecosystem damage, so large models targeting genetically modified crops should be included in high-risk types. For large models of ordinary crops, they can be classified as low-risk types. And the regulatory methods proposed by relevant departments should be made public to ensure transparency of information. Regulators can promote fairness in the deployment of agricultural large models by enforcing the use of diverse and representative data sources, which helps mitigate potential biases present in the training data (Meskó and Topol, 2023).

From the perspective of beneficence and privacy, privacy issues related to large models have received little attention or investigation in reviewed research (Yan et al., 2024). Specifically, if the training set used to train a large model contains some personal privacy information that has not been authorized by the information owner. The disregard for privacy concerns is especially worrisome considering that LLM-based innovations involve stakeholders’ natural languages, which may contain personal and sensitive information pertaining to their private lives and identities (Brown et al., 2022). If users unintentionally learn about this information while using a large model, it may cause harm to the beneficence of the information owner. Developers of large models should ensure they gain explicit consent from individuals before collecting and utilizing these personal data. Clearly communicate the purpose and scope of data usage, and offer individuals with the choice to choose out or request data deletion. Besides, limit the amount of personal and sensitive data collected and stored. Follow the principle of data minimization, ensuring that only necessary data is collected and retained. Anonymize or aggregate data whenever possible to protect individual privacy.

In general, governance approaches that promote responsible utilization of large models and focus on the outcomes rather than the technology itself will enhance research efforts and drive more innovation. By combining governance and ethics, we can harness the powerful synergy to expedite the implementation of large models in agriculture and other domains, fostering innovation at a larger scale.

## 5 Challenges and future directions

Although large models can play a powerful role in the field of agriculture, they still face challenges in many aspects.

### 5.1 Technical and practical challenges

#### 5.1.1 Difficulty in obtaining agricultural data

A primary and recurring obstacle highlighted throughout this review is the acquisition of suitable agricultural data. While large models’ data generation capabilities can partially alleviate this, as discussed in section 3.2.1, several fundamental difficulties persist:

1. **Cost and quality**: Acquiring comprehensive, high-quality, and accurately labeled real-world data is a time-consuming, labor-intensive, and costly process, especially for supervised learning approaches (Li et al., 2023a; Lu and Young, 2020).
2. **Privacy and trust**: As mentioned in section 4.2, the private nature of farmland data raises significant privacy and trust concerns among farmers, often leading to a reluctance to share information crucial for model training.
3. **Temporal complexity**: Agricultural data is inherently temporal. The need to capture entire crop growth cycles, which are influenced by daily, seasonal, and annual variations, adds another layer of complexity to data collection efforts (Li et al., 2023b).

#### 5.1.2 Low training efficiency

Directly related to the need for massive datasets is the challenge of low training efficiency and high computational cost. As systematically compared against traditional models in section 3.5 (**Figure 8**), training large agricultural models is a resource-intensive endeavor. Their massive parameter counts demand significant computational power and lengthy training times, often measured in thousands of GPU hours (Li et al., 2023b). This stands in stark contrast to the efficiency of traditional models like YOLO and Faster R-CNN, whose lower computational requirements make them a more practical and cost-effective solution for many specific, real-time agricultural tasks (Badgujar et al., 2024). This efficiency gap explains the continued prevalence of traditional models despite the emergence of more powerful large-scale architectures.

#### 5.1.3 Distribution shift

The problem of distribution shift is a major challenge when using large models in agriculture. When the data encountered by the model during deployment is obviously different from the data used in its training phase, a distribution shift will occur. The environmental conditions for collecting data may vary greatly in different regions and climates. These changes may include differences in crop types, soil conditions, weather patterns, and agricultural practices, all of which can lead to significant changes in data distribution (Wiles et al., 2021). The distribution shift will result in the trained large model not having strong applicability and may not achieve good results in some agricultural tasks. For example, it has been proven that applying large models directly to leaf segmentation tasks in a zero-shot means led to unsatisfactory performance, which can be attributed to possible distribution shifts (Chawla et al., 2021).

#### 5.1.4 The lag of data

**Figure 10:**
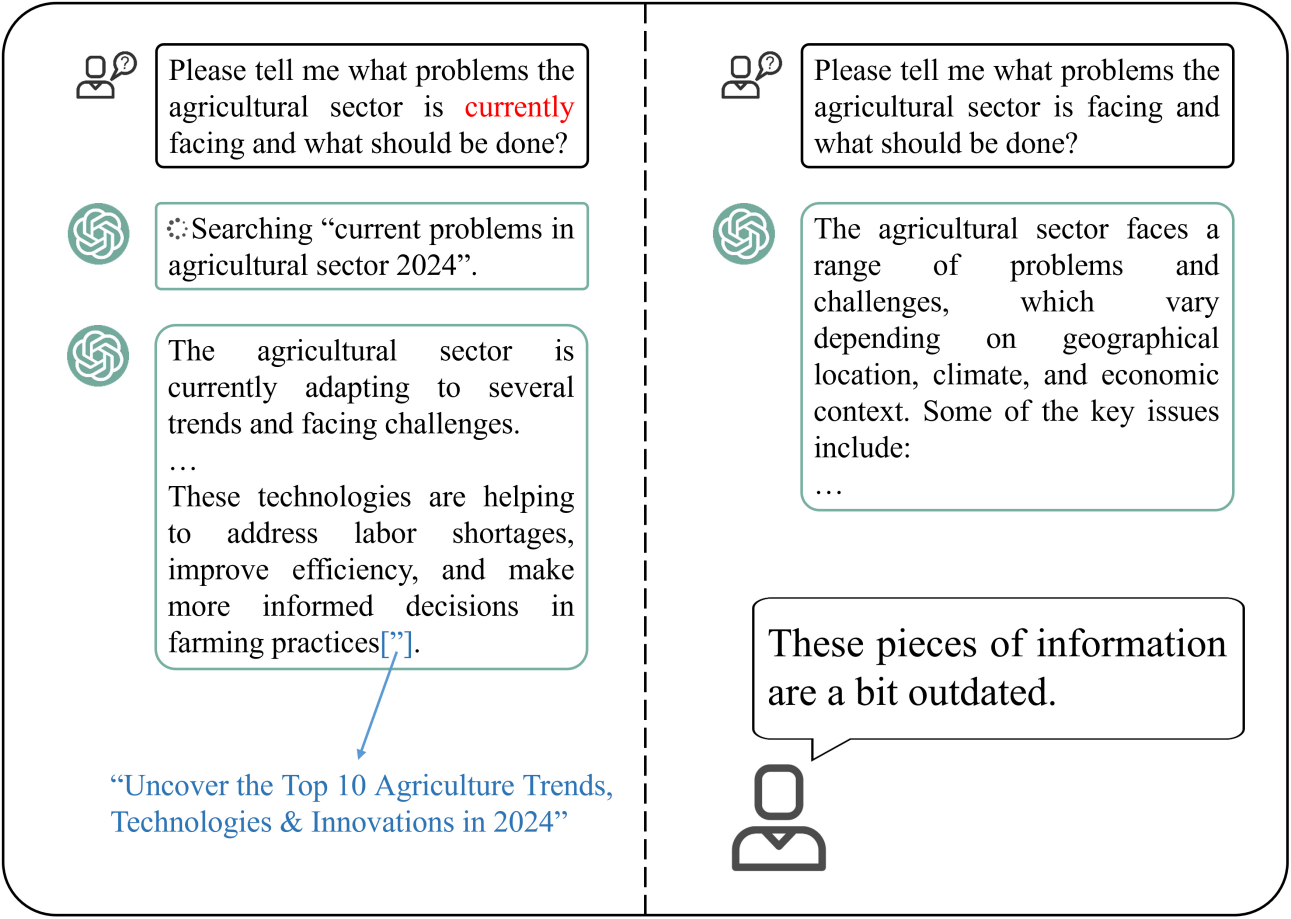
The lag of data.

After the trained large model is put into use, the data used for training has a certain timeliness for a long period of time. But after a long time, some data lags in time, and the results obtained by using a large model may deviate from the current facts (**Figure 10**).

#### 5.1.5 Query formulation impacts model output

**Figure 11:**
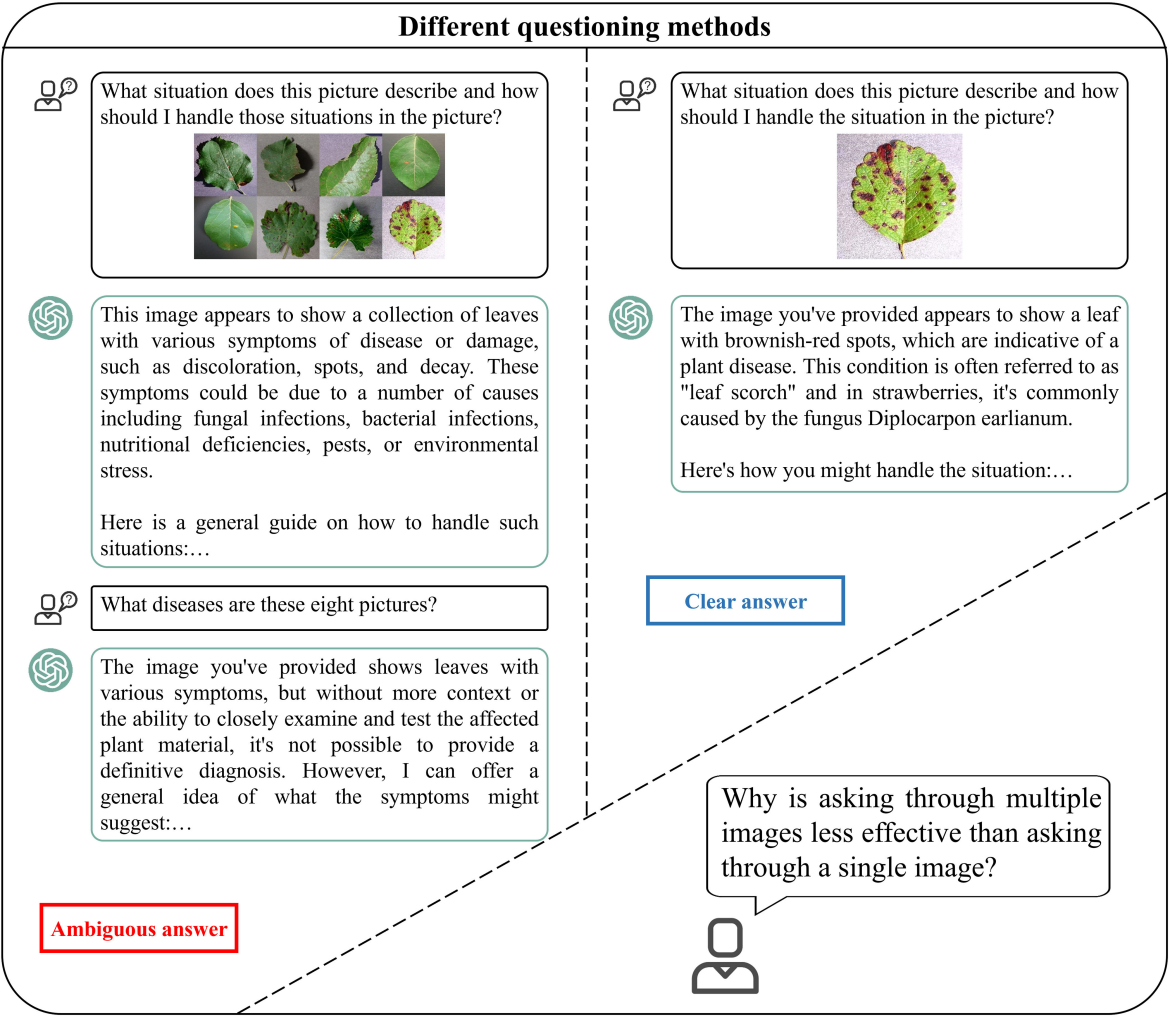
Different questioning methods can lead to different results.

The results obtained from large models can vary significantly depending on how the query is formulated. Like **Figure 11**, when multiple images are spliced together for questioning, GPT-4 provides ambiguous answers; When only asking for one image, GPT-4 provides a clearer answer.

To clear these obstacles, future research and development work needs to pay attention to model optimization techniques such as model compression and efficient network structure design, reducing model size without affecting performance (Zhong et al., 2023). It is also necessary to provide update and maintenance functions for the model to ensure its timeliness. Developers need to write relevant usage instructions to help users get started quickly. Notably, emerging frameworks like RAG offer a direct solution to the data lag and accuracy challenges by connecting LLMs to real-time, external knowledge bases. Similarly, developing more sophisticated AI Agents capable of autonomous planning and tool use will be crucial for creating robust and adaptable agricultural systems.

### 5.2 Infrastructure and cost barriers

Applying large models to rural areas faces significant barriers related to poor connectivity and high implementation costs. These limitations disproportionately affect small-scale farmers and regions with underdeveloped infrastructure, exacerbating existing inequalities in agricultural productivity and technological access (Da Silveira et al., 2023).

Dibbern et al. (2024) found that farmers often abandon digital tools due to unreliable broadband or mobile connectivity, even when initial investments are made. Technologies like IoT, cloud-based analytics, and real-time monitoring systems remain underutilized in areas lacking stable network access. This has brought some warnings for the application of large models in rural areas. In addition, the high cost of agricultural machinery using large models—render them inaccessible to resource-limited farmers. For example, autonomous machinery and AI platforms often require upfront investments exceeding $10,000 USD, a prohibitive sum for smallholders (Bolfe et al., 2020).

To overcome poor connectivity, investing in and expanding rural broadband and mobile infrastructure is crucial, potentially through government subsidies, public-private partnerships, and the exploration of alternative network solutions like satellite internet or mesh networks tailored to agricultural regions. To mitigate high implementation costs, promoting the development of affordable, modular agricultural machinery and large model platforms designed specifically for smallholder farmers is essential. In short, bridging the digital divide and promoting inclusive technological progress requires joint efforts among technology developers, agricultural researchers, policy makers, and local farmer organizations.

### 5.3 Future trends in the integration of agricultural and food sectors and large models

In the future, there will undoubtedly be agricultural large models with better performance and higher applicability. And the large models in agriculture should not be limited to text and image inputs. We believe that future multimodal agricultural models can support multimodal information such as videos (Analyzing crops in videos) and audio (Tapping watermelons, and judging maturity through the sound emitted). On the other hand, agriculture is closely related to food, and the development of large models in agriculture is likely to promote the development of large models in the food domain. Trust is indispensable for agriculture and food system technologies given food’s universality and importance to people (Tzachor et al., 2022). Researchers need to navigate complicated social, political, economic, and environmental landscapes to develop appropriate large models in the food industry. In the future food industry, researchers will strive to establish trust with governmental agencies and funders, as well as with food system partners, to provide food and products that the public trusts (Alexander et al., 2024).

Overall, although the agricultural large model still faces many challenges at present, we believe that through the joint efforts of relevant researchers in the future, these challenges can be properly addressed. And due to the close relationship between the food and agricultural domains, with the gradual development of agricultural large models, food large models will also receive further research, thereby achieving mutual positive feedback between the development of large models in these two fields.

## 6 Conclusion

In summary, this study investigated the application status of large models in the agricultural field. Our analysis establishes that these models offer unprecedented advantages through their capacity for complex reasoning, multimodal information processing, and the execution of nuanced tasks ranging from pest identification to robotic automation. We further determined that the efficacy of these powerful tools is significantly amplified when they are tailored to the agricultural domain, a crucial strategy for overcoming the pervasive challenge of limited labeled data. Furthermore, this review provided a pragmatic framework for choosing between large and traditional models, emphasizing that the decision hinges on a careful trade-off between data availability and deployment constraints. While large models excel as “generalists” in data-scarce or offline analytical scenarios, efficient traditional models remain indispensable as “specialists” for real-time, on-device tasks.

However, this vast potential is tempered by critical, interconnected challenges that must be addressed. A primary hurdle is the acquisition and utilization of suitable agricultural data; issues of data scarcity, high collection costs, inherent data diversity (across crops, regions, conditions), privacy concerns associated with farmland data, and the need for time-series information create significant obstacles. Furthermore, the high computational resources required for training and deploying large models, coupled with the often-limited internet connectivity and financial resources in rural areas, creates a significant digital divide, potentially excluding smallholder farmers. Technical issues such as model susceptibility to distribution shifts between training and deployment environments, the problem of data lag impacting real-time relevance, and sensitivity to query formulation also impact the reliability and practical applicability of current models. Finally, overarching ethical considerations, including potential biases in data or algorithms, ensuring data privacy, promoting equitable access to technology, and preventing misuse, are paramount and demand careful consideration and robust governance frameworks.

Although our study is comprehensive, there are inherent limitations to studying a rapidly developing field. To move forward, future research must directly confront the limitations and challenges identified. Developing novel techniques to mitigate data scarcity—such as advanced data augmentation and self-supervised learning tailored for agriculture—is a critical priority. Expanding multimodal capabilities to robustly incorporate inputs like video, audio, and diverse sensor data will unlock new frontiers in precision farming. Crucially, research must move beyond theoretical ethics to the practical implementation of governance structures for AI in agriculture. Furthermore, a significant opportunity lies in exploring the synergistic relationship between agricultural large models and the broader food system, addressing challenges from farm to fork.

Large models stand poised to be transformative technologies for agriculture. While significant challenges remain, the potential benefits for productivity, sustainability, and food security are immense. Addressing the technical hurdles, bridging the digital divide, and navigating the ethical landscape through collaborative, responsible innovation will be key to realizing this potential. We hope this article serves as a valuable resource and a cornerstone, stimulating further research and guiding the development of future agricultural large models that are not only powerful but also practical, efficient, and beneficial for all stakeholders in the global food system.

## Data Availability

The original contributions presented in the study are included in the article/**Supplementary Material**. Further inquiries can be directed to the corresponding authors.
